# Optimisation of the core subset for the APY approximation of genomic relationships

**DOI:** 10.1186/s12711-022-00767-x

**Published:** 2022-11-22

**Authors:** Ivan Pocrnic, Finn Lindgren, Daniel Tolhurst, William O. Herring, Gregor Gorjanc

**Affiliations:** 1 The Roslin Institute and Royal (Dick) School of Veterinary Studies, The University of Edinburgh, Easter Bush Campus, Edinburgh, EH25 9RG UK; 2 School of Mathematics, The University of Edinburgh, The King’s Buildings, Edinburgh, EH9 3FD UK; 3 Genus PIC, 100 Bluegrass Commons Blvd., Suite 2200, Hendersonville, TN 37075 USA

## Abstract

**Background:**

By entering the era of mega-scale genomics, we are facing many computational issues with standard genomic evaluation models due to their dense data structure and cubic computational complexity. Several scalable approaches have been proposed to address this challenge, such as the Algorithm for Proven and Young (APY). In APY, genotyped animals are partitioned into core and non-core subsets, which induces a sparser inverse of the genomic relationship matrix. This partitioning is often done at random. While APY is a good approximation of the full model, random partitioning can make results unstable, possibly affecting accuracy or even reranking animals. Here we present a stable optimisation of the core subset by choosing animals with the most informative genotype data.

**Methods:**

We derived a novel algorithm for optimising the core subset based on a conditional genomic relationship matrix or a conditional single nucleotide polymorphism (SNP) genotype matrix. We compared the accuracy of genomic predictions with different core subsets for simulated and real pig data sets. The core subsets were constructed (1) at random, (2) based on the diagonal of the genomic relationship matrix, (3) at random with weights from (2), or (4) based on the novel conditional algorithm. To understand the different core subset constructions, we visualise the population structure of the genotyped animals with linear Principal Component Analysis and non-linear Uniform Manifold Approximation and Projection.

**Results:**

All core subset constructions performed equally well when the number of core animals captured most of the variation in the genomic relationships, both in simulated and real data sets. When the number of core animals was not sufficiently large, there was substantial variability in the results with the random construction but no variability with the conditional construction. Visualisation of the population structure and chosen core animals showed that the conditional construction spreads core animals across the whole domain of genotyped animals in a repeatable manner.

**Conclusions:**

Our results confirm that the size of the core subset in APY is critical. Furthermore, the results show that the core subset can be optimised with the conditional algorithm that achieves an optimal and repeatable spread of core animals across the domain of genotyped animals.

**Supplementary Information:**

The online version contains supplementary material available at 10.1186/s12711-022-00767-x.

## Background

Estimating breeding values is a key operation in identifying the best animals. Estimated breeding values (EBV) are usually obtained with best linear unbiased prediction (BLUP), where genetic covariances between animals enable sharing of information between relatives and this improves the accuracy of estimation. Traditionally pedigree data have been used to construct a matrix of expected genetic covariances, $\mathbf {A}$ (the pedigree relationship matrix). With the advent of genome-wide single nucleotide polymorphism (SNP) markers, the realised genetic covariance matrix based on SNP genotypes, $\mathbf {G}$ (the genomic relationship matrix), replaced the pedigree-based $\mathbf {A}$. This change gave us the so-called genomic BLUP (GBLUP) and genomic EBV (GEBV). An essential part of estimating GEBV via Henderson’s mixed model equations is the inversion of $\mathbf {G}$ [1]. Matrix inversion has a cubic computational cost and becomes a limiting step with more than $\sim$150,000 genotyped animals [2]. A similar computational bottleneck occurred when inverting $\mathbf {A}$ with pedigree BLUP. This bottleneck was removed with a recursive algorithm of setting up the sparse inverse of $\mathbf {A}$ directly from pedigree data [3, 4]. Unfortunately, such an algorithm is not available for $\mathbf {G}$ because genome-wide SNP genotypes do not have a recursive data structure and the inverse of $\mathbf {G}$ is dense. This is an issue because the number of genotyped animals is increasing rapidly in many populations. The most remarkable case is in the US Holstein dairy cattle, where already 5 million animals have been genotyped (The Council of Dairy Cattle Breeding, https://www.uscdcb.com).

Several approaches have been proposed to solve large (single-step) GBLUP. For example, using the equivalent model with marker effects [5, 6], leveraging the matrix inversion lemma [7], using dimensionality reduction [7, 8], or inducing sparsity in the inverse through the approximation of genomic relationships [9–11]. The Algorithm for Proven and Young (APY) induces sparsity in the inverse through the approximation of genomic relationships by leveraging the limited dimensionality of genomic information in populations with a small effective population size [12]. This approximation is achieved by splitting genotyped animals into core and non-core subsets. The core animals are assumed to be all dependent on each other and hence we construct the full inverse for these animals. The non-core animals are assumed to be conditionally independent given the core animals, hence the part of the inverse for these animals is diagonal, while the part of the inverse between the core and non-core animals is dense. With APY, direct inversion is needed only for the core subset. Effectively, GEBV of the non-core animals are modelled as a function of the GEBV of the core animals. During model fitting, phenotype information “flows” between both subsets of animals, hence both subsets contribute to the estimation. While APY is an approximation, it has been shown to be accurate and scalable for various populations of dairy and beef cattle [13, 14], pigs [15, 16], and sheep [17, 18].

There are two crucial decisions in APY. First, how many animals should form the core subset. Second, which animals should form the core subset. Empirical testing suggests that the optimal number of core animals is connected to the effective population size and the dimensionality of genomic information [12]. As such, the optimal number of core animals can be gauged by the number of eigenvectors that explain a large percentage of variation in SNP genotypes (or equivalently in $\mathbf {G}$) [19]. For example, by setting the number of core animals to the number of eigenvectors that captured more than 98% of the variation in $\mathbf {G}$, the GEBV were comparable to those obtained with the full $\mathbf {G}$. Empirical testing also suggests that a random choice of core animals gives satisfactory accuracy of GEBV [20]. However, a random choice is not desired in routine genetic evaluations because it could result in small changes in GEBV, possibly reranking animals, even with the same data. A recent study suggested that such changes are a natural part of genetic evaluations when the data is updated [21]. Still, genetic evaluations should return the same GEBV with the same data. An extensive study on size and definitions of core subsets in a pig dataset [22] showed that their definition matters for small core sizes. Therefore, there is a need for methods that construct an optimal core subset that maximises accuracy of GEBV and ensures stable results between different model runs.

The aim of this paper is to present and evaluate alternative core subset constructions for APY. First, we propose a novel algorithm for optimising the core subset based on the conditional genomic relationship matrix or the conditional SNP genotype matrix. We termed this method as the conditional core subset algorithm, or simply conditional algorithm. Second, we compared the conditional core subset construction with other core subset constructions using a simulated cattle dataset and a real pig dataset.

## Methods

We analysed how different core subset sizes and different core subset constructions affect the accuracy of GEBV with APY. Specifically, we compared core subset construction (1) at random; (2) based on the diagonal of the genomic relationship matrix; (3) at random with weights from (2); or (4) based on the conditional algorithm. We compared the different core subset constructions with the number of core animals set to the number of largest eigenvalues that captured 10, 30, 50, 70, 90, 95, 98, and 99% of the variation in $\mathbf {G}$. To demonstrate the functionality of different core subset constructions, we used a small simulated cattle dataset. To demonstrate their practical utility, we used a large real pig dataset. In the following, we first describe the simulated and real datasets as well as the models fitted to each dataset. We then describe different core subset constructions for APY, including the derivation of the conditional algorithm. Finally, we describe the validation of GEBV and data visualisation.

### Simulated cattle data

We simulated a simple breeding programme using the AlphaSimR R package [23]. The simulated genome consisted of 10 chromosomes with 1100 segregating sites per chromosome (11,000 in total), from which we randomly assigned 100 sites (1000 in total) as additive quantitative trait loci (QTL), and 1000 sites (10,000 in total) as SNP markers. There was no overlap between QTL and SNPs. The historical effective population size followed cattle estimates [24], with a mutation rate of $2.5\times 10^{-8}$, and a recombination rate of $1.0\times 10^{-8}$. Phenotypes were assumed to have a heritability of 0.30. From the initial founder population of 3000 animals, we randomly selected 1500 females and 50 males to serve as parents of the first generation. In each succeeding generation, 3000 animals with equal female to male sex ratio were obtained from mating 1500 females and 50 males, with a fixed number of two offspring per female. Females were replaced at a rate of 50%, meaning that the 750 best young females out of 1500 and the 750 best old females out of 1500 were dams of the next generation. This dam selection was based on their phenotype value. On the male side, the best 45 young males out of 1500 and the best 5 old males out of 45 were sires of the next generation. This sire selection was based on their true genetic value (mimicking accurate selection). SNP genotypes were collected for all animals in generations 15 to 20, resulting in 15,000 genotyped and phenotyped animals in our study. The 3000 genotyped animals from generation 20 served as a validation subset.

#### Simulated cattle data analysis

We analysed the simulated phenotype data and SNP genotype data with GBLUP:

$$\begin{aligned} \mathbf {y} = \mathbf {1}\mu + \mathbf {Z}_a\mathbf {a} + \mathbf {e}, \end{aligned}$$

where $\mathbf {y}$ is a vector of phenotypes, $\mathbf {1}$ is a vector of 1s, $\mu$ is the overall mean, $\mathbf {Z}_{a}$ is a design matrix connecting phenotypes to animal breeding values $\mathbf {a}$, and $\mathbf {e}$ is a vector of residuals. We assumed that $\mathbf {a} \sim N\left( \mathbf {0}, \mathbf {G}\sigma ^2_a\right)$ and $\mathbf {e} \sim N\left( \mathbf {0}, \mathbf {I}\sigma ^2_e\right)$, where $\mathbf {G}$ is the genomic relationship matrix, $\sigma ^2_a = 1.00$ is the variance of breeding values, and $\sigma ^2_e = 2.33$ is the variance of residuals.

### Real pig data

Phenotypic data for a moderately heritable trait measured from 1999 to 2021 on 42,868 pigs (33,544 purebred—L1, 114 crossbred—F1 (L1 × L2), and 9210 backcross—BC1 (L1 × F1) and BC2 (L1 × BC1)) were provided by PIC (a Genus company, Hendersonville, TN, USA). Data on purebred L2 were not available in this study. SNP genotypes were available for 42,707 SNPs after quality control. The number of genotyped pigs was 49,788 (37,598 purebred—L1, 486 crossbred—F1, and 11,704 backcross—BC1 and BC2). The validation subset included 478 phenotyped and genotyped youngest animals (L1 and BC2) born in 2021, with their phenotypes removed from the analysis.

#### Real pig data analysis

We analysed the real phenotype data and SNP genotype data with GBLUP:

$$\begin{aligned} \mathbf {y} = \mathbf {Xb} + \mathbf {Z}_a\mathbf {a} + \mathbf {Z}_l\mathbf {l} + \mathbf {e}, \end{aligned}$$

where $\mathbf {y}$ is a vector of phenotypes, $\mathbf {X}$ is a design matrix connecting phenotype to contemporary group fixed effects $\mathbf {b}$, $\mathbf {Z}_a$ is a design matrix connecting phenotypes to animal breeding values $\mathbf {a}$, $\mathbf {Z}_l$ is a design matrix connecting phenotypes to shared litter effect $\mathbf {l}$, and $\mathbf {e}$ is a vector of residuals. We assumed that $\mathbf {a} \sim N\left( \mathbf {0}, \mathbf {G}\sigma ^2_a\right)$, $\mathbf {c} \sim N\left( \mathbf {0}, \mathbf {I}\sigma ^2_l\right)$, and $\mathbf {e} \sim N\left( \mathbf {0}, \mathbf {I}\sigma ^2_e\right)$, where $\mathbf {G}$ is the genomic relationship matrix, $\sigma ^2_a$ is the variance of breeding values, $\sigma ^2_l$ is the variance of litter effects, and $\sigma ^2_e$ is the variance of residuals.

### Genomic relationship matrix and APY inverse

For both simulated and real datasets, the genomic relationship matrix was calculated as $\mathbf {G}= \mathbf {W}\mathbf {W}^{{\!\scriptscriptstyle \top }} / 2\sum ^{n_m}_{j=1}{p_j(1-p_j)}$, where $\mathbf {W}$ is a centred matrix of SNP genotypes (coded as 0 for the reference homozygote, 1 for the heterozygote, and 2 for the alternative homozygote), $p_j$ is the observed frequency of the alternative allele for SNP *j*, and $n_m$ is the number of SNPs [25]. To ensure $\mathbf {G}$ is positive definite, $\mathbf {G}$ was blended with $0.01\mathbf {I}$, where $\mathbf {I}$ is the identity matrix. The final $\mathbf {G}$ was either inverted directly or with APY.

The APY partitions $\mathbf {G}$ into:

$$\begin{aligned} \mathbf {G} = \left[ \begin{array}{ll}\mathbf {G}_{cc}&{}\mathbf {G}_{cn}\\ \mathbf {G}_{nc}&{}\mathbf {G}_{nn}\\ \end{array}\right] , \end{aligned}$$

where *c* and *n* correspond to the core and non-core subsets. The APY inverse [9] is then:

$$\begin{aligned} \mathbf {G}_{\text {APY}}^{-1} = \left[ \begin{array}{ll}\mathbf {G}_{cc}^{-1}&{}\mathbf {0}\\ \mathbf {0}&{}\mathbf {0}\\ \end{array}\right] +\left[ \begin{array}{ll}{-\mathbf {G}}_{cc}^{-1}\mathbf {G}_{cn}\\ \mathbf {I}\\ \end{array}\right] \mathbf {M}_{nn}^{-1}\left[ {-\mathbf {G}}_{nc}\mathbf {G}_{cc}^{-1} \ \ \mathbf {I}\right] , \end{aligned}$$

where $\mathbf {M}_{nn}$ is a diagonal matrix with non-zero elements given by $\mathbf {M}_{nn,ii}=g_{ii}-\mathbf {g}_{ic}\mathbf {G}_{cc}^{-1}\mathbf {g}_{ci}$ in which $g_{ii}$ is the $i^{th}$ diagonal element of $\mathbf {G}_{nn}$ and $\mathbf {g}_{ci} = \mathbf {g}_{ic}^{{\!\scriptscriptstyle \top }}$ is the $i^{th}$ column of $\mathbf {G}_{cn}$ (or the $i^{th}$ row of $\mathbf {G}_{nc}$). This formulation induces a sparse inverse that approximates the full inverse. Here we only need the direct inverse of the core matrix $\mathbf {G}_{cc}$ and the diagonal matrix $\mathbf {M}_{nn}$.

We defined the core and non-core subsets by splitting the genotyped animals. The number of core animals was the same across all evaluated core subset constructions. We matched the number of core animals with the number of eigenvectors that captured 10, 30, 50, 70, 90, 95, 98, and 99% of variation in $\mathbf {G}$ [19]. The remaining genotyped animals then formed the non-core subset.

Instead of eigenvalue decomposition of $\mathbf {G} \ (n\times n)$, we used an equivalent singular value decomposition (SVD) of $\mathbf {W} \ (n\times n_{m})$, because SVD has a $O(n_{m}^2n)$ computational cost, while eigenvalue decomposition has a $O(n^3)$ computational cost. The SVD is given by $\mathbf {W}=\mathbf {UDV}^{{\!\scriptscriptstyle \top }}$, where $\mathbf {D}$ is a diagonal matrix of singular values that correspond to the square root of the non-zero eigenvalues of $\mathbf {W}^{{\!\scriptscriptstyle \top }}\mathbf {W}$ and $\mathbf {W}\mathbf {W}^{{\!\scriptscriptstyle \top }}$. The columns of $\mathbf {U}$ are left singular vectors that correspond to the eigenvectors of $\mathbf {W}\mathbf {W}^{{\!\scriptscriptstyle \top }}$, such that $\mathbf {U}^{{\!\scriptscriptstyle \top }}\mathbf {U}=\mathbf {I}$. The columns of $\mathbf {V}$ are right singular vectors that correspond to the eigenvectors of $\mathbf {W}^{{\!\scriptscriptstyle \top }}\mathbf {W}$, such that $\mathbf {V}^{{\!\scriptscriptstyle \top }}\mathbf {V}=\mathbf {I}$. The eigenvalue decomposition of $\mathbf {G}$ is then obtained as $\mathbf {G} = \mathbf {W}\mathbf {W}^{{\!\scriptscriptstyle \top }}/v = \mathbf {UD}^2\mathbf {U}^{{\!\scriptscriptstyle \top }}/v$, where $v=2\sum _{j=1}^{n_m}{p_j(1-p_j)}$. The SVD of $\mathbf {W}$ was computed with the base R function svd [26] for the simulated data and a Fortran program using LAPACK subroutine DGESVD [27] for the real pig data.

### Core subset constructions

A guiding principle in APY is to construct a core subset in such a way that the APY inverse approximates the full inverse $\mathbf {G}^{-1}$ well. This can be achieved by a sufficiently large core subset. Furthermore, for a given size of a core subset, we want to cover as much variation in the SNP genotype matrix (and hence in $\mathbf {G}$) as possible. To achieve this, we used four different core subset constructions. The first construction (random), used randomly sampled genotyped animals to form the core. This construction is the most common in current APY applications and has hence served as a baseline. The second construction (diagonal), chose core animals based on the diagonal elements in $\mathbf {G}$ [17]. The principle behind this construction is that animals with large diagonal elements in $\mathbf {G}$ deviate substantially from the centroid of SNP genotypes and therefore sample variation in SNP genotypes well. An alternative view of this principle is to recognise that the trace of a matrix is equal to the sum of its eigenvalues. Hence, selecting animals with the largest diagonal elements in $\mathbf {G}$ maximises the amount of captured variation. However, this construction can oversample individuals from the most inbred families, while we would like to cover as many families as possible for a given core subset size. The third construction (weighted), was a combination of the first two approaches, where the core animals were randomly sampled, but with a weight based on the corresponding diagonal element in $\mathbf {G}$. The weighted construction attempted to alleviate the potential oversampling issue with the diagonal construction. We used the R function sample_n [28] with option weight for weighted sampling. For the random approaches (random and weighted), sampling was replicated five times to manifest potential variability in GEBV with a random core subset. The fourth construction (conditional), was based on a conditional algorithm inspired by sequential sampling, e.g. [29, 30], which we describe in the following.

The principle behind the conditional algorithm is to spread core animals across the domain of genotyped animals or equivalently the domain of collected SNP genotype data. This is achieved by choosing animals far away from each other in the covariance sense. The algorithm initiates the core subset with the animal that deviates the most from the centroid of SNP genotypes. Then, it sequentially finds animals that deviate the most from the core animal(s). By growing the core subset, the algorithm optimally samples the domain.

Before we introduce the algorithm, we will show how to find animals that are far away from each other in the covariance sense. We start with the joint covariance matrix $\mathbf {C}=\mathbf {WW}^{\!\scriptscriptstyle \top }$, where the variance for animal *i* is the squared norm of the *i*-th row of $\mathbf {W}$, or equally the *i*-th diagonal element of $\mathbf {WW}^{{\!\scriptscriptstyle \top }}$. For simplicity the scaling constant is omitted, so $\mathbf {G}=\mathbf {C}/v$. Let $\mathbf {w}$ denote the vector corresponding to the *i*-th row of $\mathbf {W}$ (SNP genotypes of the animal *i*). Let $\mathbf {e}$ denote a “selector” vector of 0s and a single 1 at position *i*, so that $\mathbf {w}=\mathbf {e}^{{\!\scriptscriptstyle \top }}\mathbf {W}$, $\mathbf {C}_{i,i}=\mathbf {e}^{{\!\scriptscriptstyle \top }}\mathbf {C}\mathbf {e}$, and $\mathbf {C}_{i,.}=\mathbf {e}^{{\!\scriptscriptstyle \top }}\mathbf {C}$. To find animals that are far away from each other in the covariance sense we use the conditional covariance matrix $\mathbf {C}_{cond}$, where we condition on core animals. Specifically, large diagonals of $\mathbf {C}_{cond}$ indicate animals whose SNP genotypes are not well represented by the SNP genotypes of core animals. We implemented the algorithm by expanding the core subset one animal at a time. Hence, we require the conditional covariance matrix given animal *i*, which is:

$$\begin{aligned} \mathbf {C}_{cond}&= \mathbf {C} - \mathbf {C}\mathbf {e}\left( \mathbf {e}^{{\!\scriptscriptstyle \top }}\mathbf {C}\mathbf {e}\right) ^{-1}\mathbf {e}^{{\!\scriptscriptstyle \top }}\mathbf {C} \\&= \mathbf {WW}^{{\!\scriptscriptstyle \top }} - \mathbf {WW}^{{\!\scriptscriptstyle \top }}\mathbf {e}\left( \mathbf {e}^{{\!\scriptscriptstyle \top }}\mathbf {WW}^{{\!\scriptscriptstyle \top }}\mathbf {e}\right) ^{-1}\mathbf {e}^{{\!\scriptscriptstyle \top }}\mathbf {WW}^{{\!\scriptscriptstyle \top }} \\&= \mathbf {WW}^{{\!\scriptscriptstyle \top }}-\mathbf {Ww}^{{\!\scriptscriptstyle \top }}\left( \mathbf {ww}^{{\!\scriptscriptstyle \top }}\right) ^{-1}\mathbf {wW}^{{\!\scriptscriptstyle \top }}. \end{aligned}$$

Since $\mathbf {ww}^{{\!\scriptscriptstyle \top }}$ is the squared norm ($||\mathbf {w}||^{2}$) of the row vector $\mathbf {w}$, we introduce a normalised vector $\mathbf {a}$, where $\mathbf {a}=\frac{\mathbf {w}}{\left| \mathbf {w}\right| }$, $\mathbf {a}^{{\!\scriptscriptstyle \top }}\mathbf {a}=\mathbf {w}^{{\!\scriptscriptstyle \top }}\left( \mathbf {ww}^{{\!\scriptscriptstyle \top }}\right) ^{-1}\mathbf {w}$, and $\mathbf {aa}^{{\!\scriptscriptstyle \top }}=1$. By using $\mathbf {a}$, we can shorten the expression for the conditional covariance to:

$$\begin{aligned} \mathbf {C}_{cond}=\mathbf {WW}^{{\!\scriptscriptstyle \top }}-\mathbf {Wa}^{{\!\scriptscriptstyle \top }}\mathbf {aW}^{{\!\scriptscriptstyle \top }}. \end{aligned}$$

By adding and subtracting $\mathbf {Wa}^{{\!\scriptscriptstyle \top }}\mathbf {aW}^{{\!\scriptscriptstyle \top }}$ we can factorise the conditional covariance as:

$$\begin{aligned} \mathbf {C}_{cond}&= \mathbf {WW^{{\!\scriptscriptstyle \top }}-Wa^{{\!\scriptscriptstyle \top }}aW^{{\!\scriptscriptstyle \top }}-Wa^{{\!\scriptscriptstyle \top }}aW^{{\!\scriptscriptstyle \top }}+Wa^{{\!\scriptscriptstyle \top }}aW^{{\!\scriptscriptstyle \top }}} \\&= \mathbf {WW^{{\!\scriptscriptstyle \top }}-Wa^{{\!\scriptscriptstyle \top }}aW^{{\!\scriptscriptstyle \top }}-Wa^{{\!\scriptscriptstyle \top }}aW^{{\!\scriptscriptstyle \top }}+Wa^{{\!\scriptscriptstyle \top }}aa^{{\!\scriptscriptstyle \top }}aW^{{\!\scriptscriptstyle \top }}} \\&= \mathbf {(W-Wa^{{\!\scriptscriptstyle \top }}a)(W-Wa^{{\!\scriptscriptstyle \top }}a)^{{\!\scriptscriptstyle \top }}}. \end{aligned}$$

We can calculate the conditional SNP genotype matrix (conditional on the SNP genotypes of animal *i*) as:

$$\begin{aligned} \mathbf {W}_{cond}=\mathbf {W}-\mathbf {Wa}^{{\!\scriptscriptstyle \top }}\mathbf {a}. \end{aligned}$$

Therefore, the conditional covariance matrix given animal *i* is:

$$\begin{aligned} \mathbf {C}_{cond}=\mathbf {W}_{cond}\mathbf {W}^{{\!\scriptscriptstyle \top }}_{cond}. \end{aligned}$$

The conditional algorithm (Algorithm 1) starts with a desired size of the core subset, $n_c$, an empty vector to store core animals, $\mathbf {k}$, and the joint covariance matrix, $\mathbf {C}_0 = \mathbf {W}\mathbf {W}^{{\!\scriptscriptstyle \top }}$. The algorithm iterates for $n_c$ rounds by sequentially choosing core animals based on their conditional variance. In the *i*-th round, it expands the core subset with an animal that has the largest conditional variance in $\mathbf {C}_{i-1}$, that is, conditional on the $(i-1)$ previously chosen core animals. Then it updates $\mathbf {C}_{i-1}$ to $\mathbf {C}_{i}$ by conditioning on the currently chosen core animal. In the next round, the updated conditional covariance matrix $\mathbf {C}_{i}$ is used to choose the next core animal, and so on.
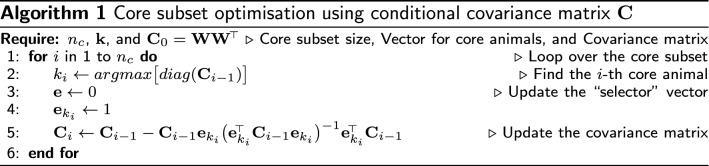


Forming and repeatedly updating a large covariance matrix can be demanding in terms of computation and storage. The conditional algorithm can work with the SNP genotype matrix instead of the covariance matrix by using the expression for the conditional SNP genotype matrix, $\mathbf {W}_{cond}$ (Algorithm 2). In line 2 of this algorithm, we show the expression $diag(\mathbf {W}_{i-1}\mathbf {W}_{i-1}^{\!\scriptscriptstyle \top })$, which forms the covariance matrix and extracts its diagonal. We can speedup this step by calculating only the diagonal (conditional variance) by squaring and summing every row of $\mathbf {W}_{i-1}$.
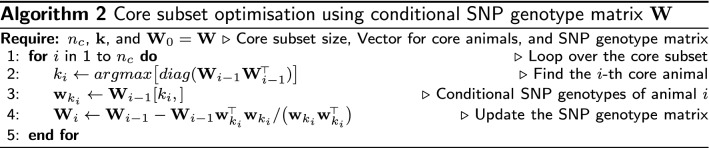


The above presented algorithm does not guarantee a globally optimal core subset. Namely, a different starting core animal will lead sequential updates to a different core subset. Achieving global optimality is demanding. To partially address the optimality issue, we have extended the algorithm to choose core animals that sequentially minimise conditional variances of other animals. That is, choosing a core animal that captures as much variation in SNP genotypes of other animals as possible. This principle follows closely the principle of APY approximation. We have derived two versions of the extension by sequentially minimising the maximum or average conditional variance of other animals. To implement the extended algorithm, we have to replace line 2 of Algorithm 2 with line 4 and run this updated line for every animal. Unfortunately, the addition of this inner loop makes the extended algorithm impractically slow and we have not tested it further.

### Analyses

We compared the different core subset constructions using the correlation between GEBV obtained with the full inverse and GEBV obtained with the APY inverse. Furthermore, for the simulated data we assessed the accuracy for validation animals as the correlation between their GEBV and true breeding values (TBV). For the real pig dataset, we assessed the accuracy for validation animals as the correlation between their GEBV and phenotypes adjusted for the fixed effects in the model. We consider the accuracy with the full $\mathbf {G}$ inverse as the baseline. Furthermore, we assessed the dispersion bias for validation animals as the regression of their phenotypes adjusted for the fixed effects on their GEBV. In this sense, a regression coefficient smaller or greater than 1 indicates the GEBV are either inflated or deflated, respectively.

To gain insight into where the chosen core animals are positioned with regards to each other, we have visualised the population structure of genotyped animals with dimension reduction techniques. We used the classical linear Principal Component Analysis (PCA) and the novel non-linear Uniform Manifold Approximation and Projection (UMAP; [31]). We obtained UMAP using the umap R package [32] with default parameters.

### Computations

The simulated cattle data was analysed within the R environment [26], including the construction of genomic relationship matrices and their inverses by direct or APY inversion, and by calling the BLUPF90 software [33] to solve the mixed model equations. Due to the size, the mixed model equations corresponding to the real pig dataset were solved by a preconditioned conjugate gradient algorithm as implemented in the BLUP90IOD2 software [34] with the convergence criterion set to $10^{-12}$ and executed on The University of Edinburgh High-Performance Computing environment (Edinburgh Compute and Data Facility; http://www.ecdf.ed.ac.uk). For the simulated and real data sets, we assumed that the variance components were known and not estimated. All figures were produced using ggplot2 [35] and VennDiagram [36] R packages. The code for the simulation, as well as for core subset construction and genetic evaluation, is available from https://github.com/HighlanderLab/ipocrnic_OptimisedCore4APY and https://doi.org/10.5281/zenodo.7181323.

## Results

The quality of the APY approximation depends on the size of the core subset. For a given core subset size we can optimise its construction to ensure stability of GEBV. We show this by calculating the correlations between the GEBV obtained with the full inverse and GEBV obtained with the APY inverses, as well as the respective validation accuracies. By plotting the population structure with PCA and UMAP, we show how the conditional algorithm spreads core animals far away from each other in the covariance sense. Instability of the random construction is illustrated by Venn diagrams of overlapping core animals between replicates. We present these results separately for the simulated cattle dataset and the real pig dataset.

### Simulated cattle data

For the simulated dataset, the number of core animals was 10, 50, 135, 326, 968, 1516, 2386, and 3129. These core subset sizes corresponded to the number of largest eigenvalues that captured 10, 30, 50, 70, 90, 95, 98, and 99% of the variation in $\mathbf {G}$.

**Fig. 1:**
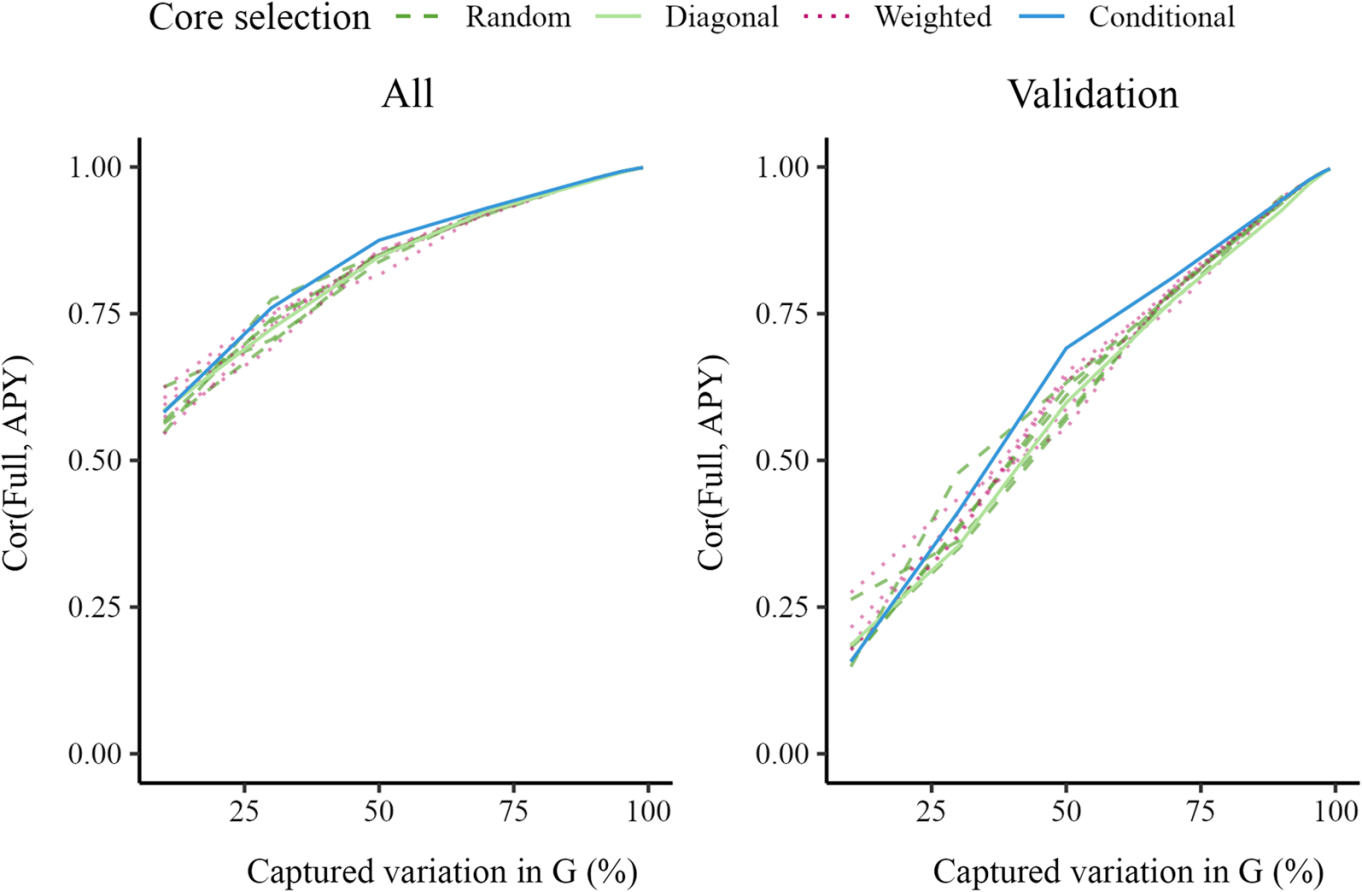
Correlations between $\mathbf {GEBV_{Full}}$ and $\mathbf {GEBV_{APY}}$ for all animals and only validation animals in simulation. Genomic estimated breeding values (GEBV) were based on the full inverse (Full) or the Algorithm for Proven and Young inverse (APY) of the genomic relationship matrix ($\mathbf {G}$). For APY, the core subset was constructed at random (Random), based on the highest diagonal in $\mathbf {G}$ (Diagonal), a combination of Random and Diagonal (Weighted), or the conditional algorithm (Conditional). Random and weighted constructions show five samples

Figure 1 shows correlations between GEBV obtained with the full inverse and GEBV obtained with the APY inverse, calculated either for the whole genotyped or validation population. The correlations increased with increasing core subset for all four core subset constructions. The conditional construction had generally the highest correlation. Other constructions had comparable correlations. The correlation calculated for the validation population was more sensitive to core subset size than the correlation for the whole population. The correlations were greater than 0.99 when the number of core animals corresponded to the number of largest eigenvalues that captured 98% of the variation in $\mathbf {G}$. There was considerable variation in correlation between replicates with the random and weighted constructions, although this variation decreased as the core subset increased. With a large core subset (2386 core animals), the correlations between five consecutive replicates of 3000 validation animals were all equal to 0.99, for random and weighted constructions.

**Fig. 2:**
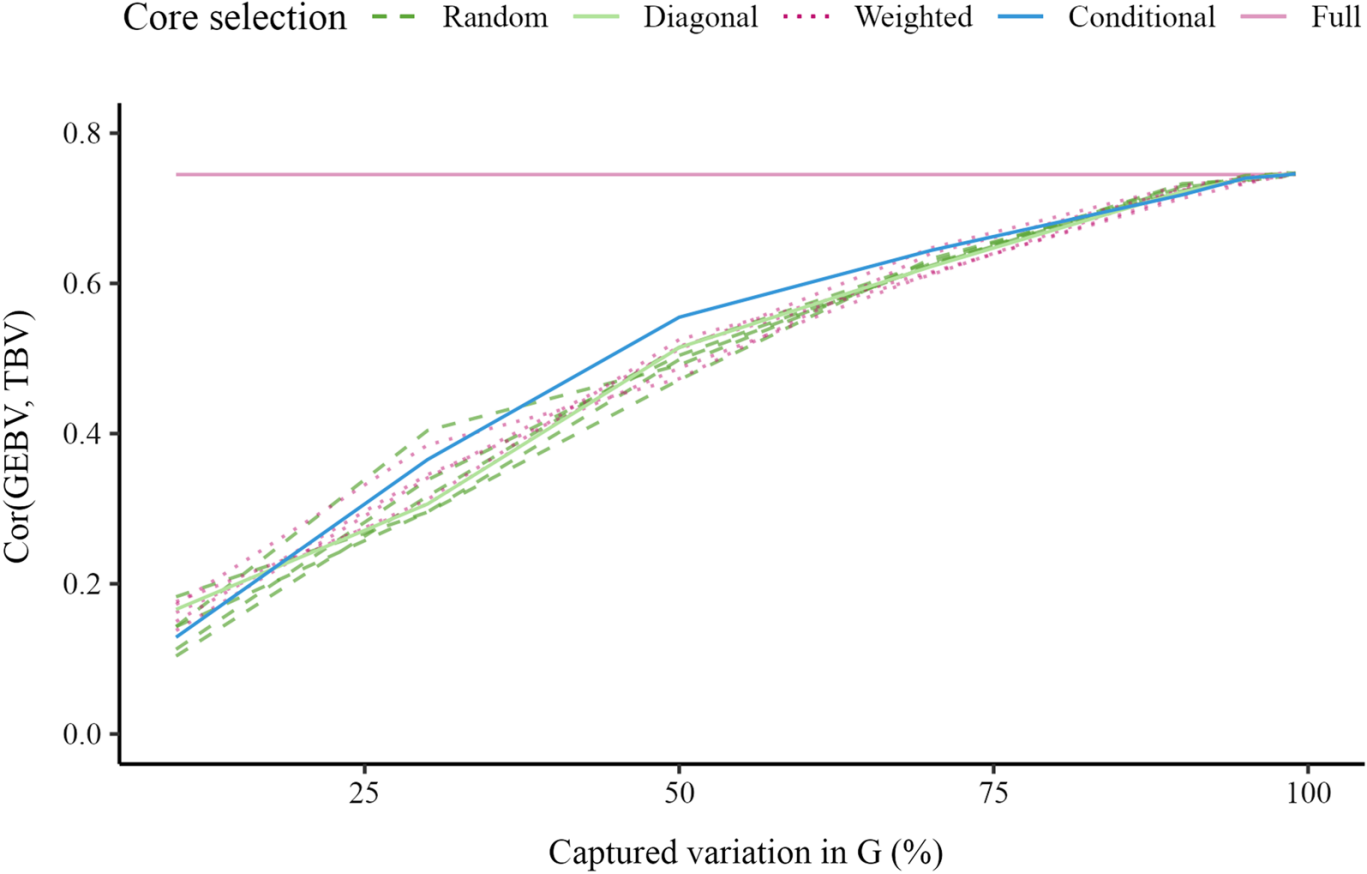
Accuracy for validation animals in simulation. Accuracy is the correlation between genomic estimated breeding values (GEBV) and true breeding values (TBV) from simulation. GEBV were based on the full inverse (Full) and the Algorithm for Proven and Young (APY) inverse of the genomic relationship matrix ($\mathbf {G}$). For APY, the core subset was constructed at random (Random), based on the highest diagonal in $\mathbf {G}$ (Diagonal), a combination of Random and Diagonal (Weighted), or the conditional algorithm (Conditional). Random and weighted constructions show five samples

Figure 2 presents the accuracy of GEBV for validation animals as a function of the percentage of captured variation in $\mathbf {G}$. The accuracy with the full inverse was 0.74, which represents the baseline. The accuracy with the APY inverse increased from 0.10 to 0.75 as the core subset increased. There were only minimal differences between the four core subset constructions in accuracy trends, but we note the variation in accuracy with random constructions and that conditional construction generally had the largest correlation. The accuracy reached or even marginally surpassed (for about 0.001), the accuracy obtained with the full inverse, when the number of core animals corresponded to the number of largest eigenvalues that captured 98% of the variation in $\mathbf {G}$.

To understand how the different core subset constructions may affect selection decisions, we compared the top 3% (45/1500) of young males selected based on the GEBV from the full inverse or the APY inverse. These selected young males were used as sires of the next generation so their ranking is important. When the core subset corresponded to the number of largest eigenvalues that captured 98% of the variation in $\mathbf {G}$, very similar animals were selected using GEBV calculated with the full inverse compared to the APY inverse. Specifically, compared to the 45 young males selected based on the GEBV from the full inverse, the random construction selected 38±2 of the same young males, the diagonal construction selected 37 of the same young males, the weighted construction selected 39±1 of the same young males, and the conditional construction selected 39 of the same young males. Therefore, random construction had somewhat more ranking variation than weighted construction. There is no variation in diagonal and conditional constructions by design.

Compared to the random construction, the conditional construction could be more time consuming because the conditional algorithm sequentially updates a large SNP genotype matrix, $\mathbf {W}$. To reduce computation time, we also ran the conditional algorithm on a reduced rank SNP genotype matrix obtained via $\mathbf {W}_r=\mathbf {U}_r\mathbf {D}_r$, where $\mathbf {U}_r\mathbf {D}_r$ correspond to the first *r* principal components of $\mathbf {W}$ that captured 99% of the variation in $\mathbf {G}$, that is 3130 of the 15,000 principal components. Using the reduced rank SNP genotype matrix $\mathbf {W}_r$ (with conditional construction) produced very similar accuracies of the GEBV as using the full SNP genotype matrix $\mathbf {W}$ (see Additional file 1). However, there were some differences between the chosen core animals when using $\mathbf {W}_r$ or $\mathbf {W}$, which increased proportionally with the number of core animals. For example, with 10 core animals the overlap was 90% (9/10), with 135 it was 86% (116/135), but with 2386 it was only 71% (1696/2386). Lastly, note that the time required to choose core animals using reduced rank $\mathbf {W}_r$, to obtain the APY inverse in R, to read the inverse and solve the mixed model equations in BLUPF90, was reduced by more than half compared to using the full $\mathbf {W}$.

**Fig. 3:**
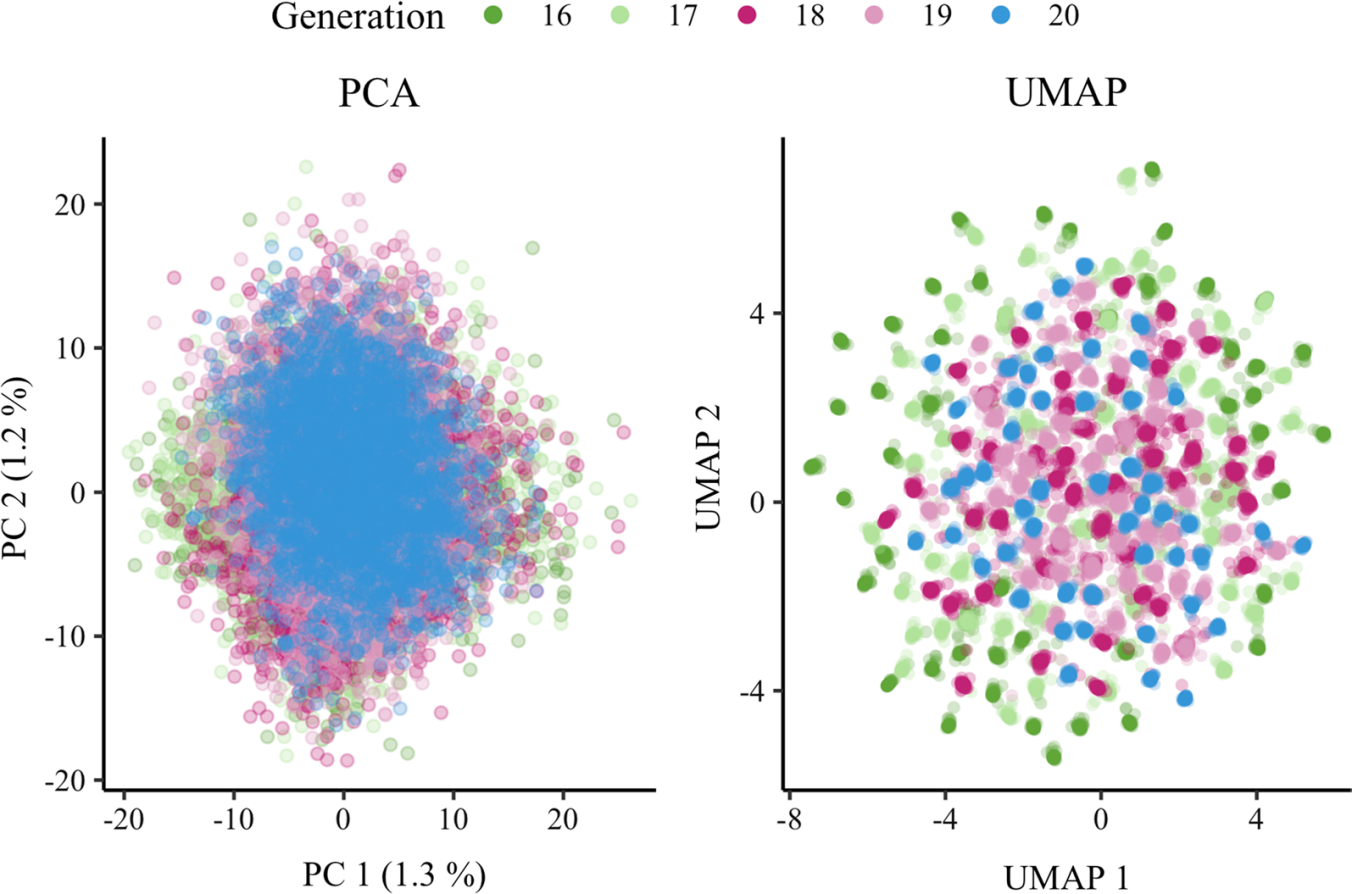
Visualisation of PCA and UMAP for genotyped animals in simulation. Projection of genomic relationships into the first two dimensions was done with Principal Components Analysis (PCA) or with Uniform Manifold Approximation and Projection (UMAP). The percentage of variation captured by each principal component is shown in parentheses. Colours represent five different generations of genotyped animals in simulation

**Fig. 4:**
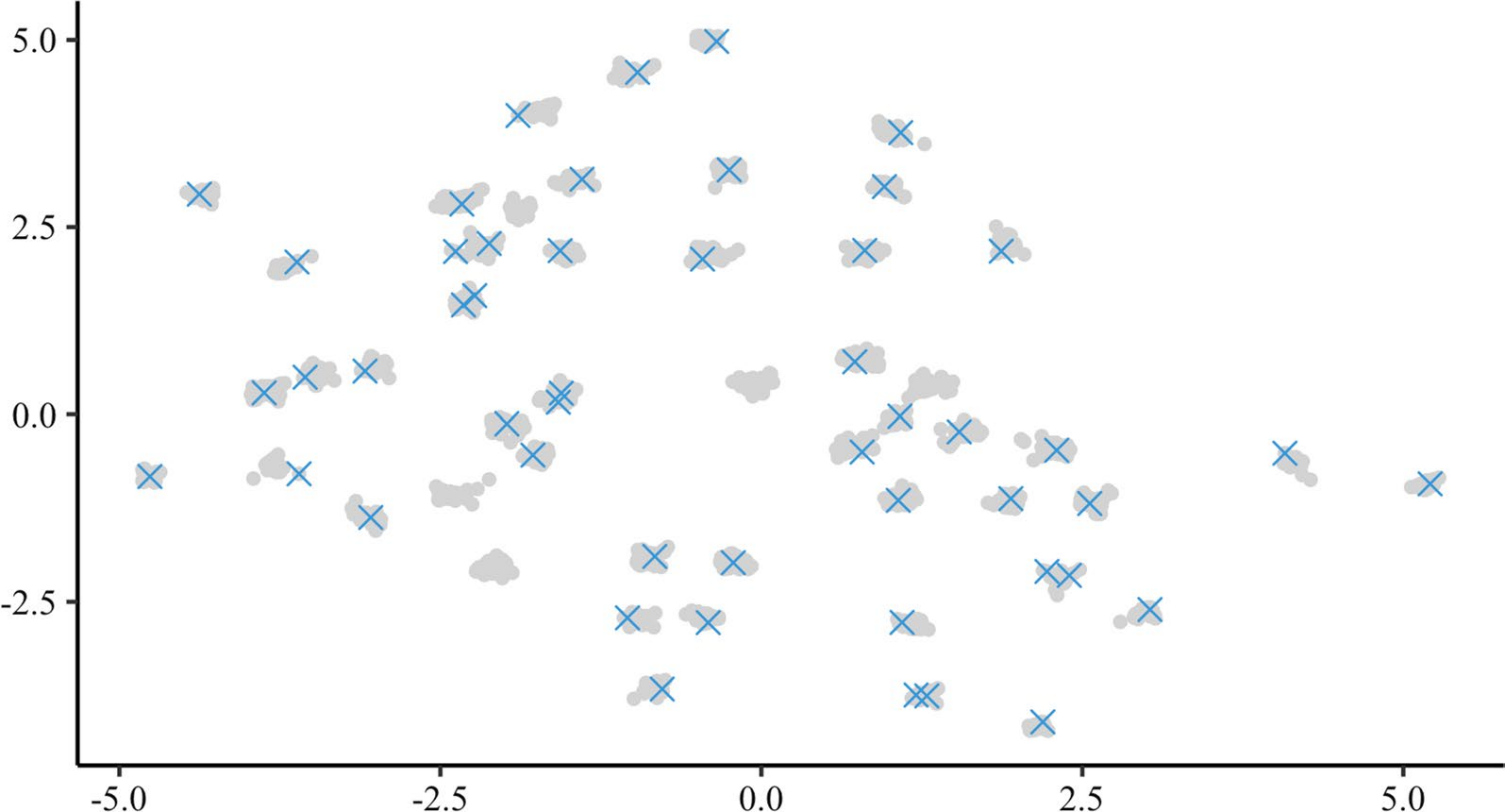
Spread of core animals in the last generation of simulation. Visualisation of the Uniform Manifold Approximation and Projection (UMAP) for animals (gray dots) in the last generation of simulation representing 50 paternal half-sib families. Overlaid are 50 core animals chosen by the conditional algorithm (blue crosses)

Figure 3 shows PCA and UMAP for the 15,000 animals in simulation, with data points coloured by generation. While both methods captured general homogeneity of the simulated population, UMAP also revealed fine-scale population structure and clearly highlighted a change in variation between generations (see Additional file 2). Namely, the projected points moved closer as the generation number increased. This trend suggests loss of genetic variation due to selection and the creation of distinct clusters of paternal half-sib families. For example, the 50 clusters identified by UMAP in the validation set (generation 20) correspond to exactly 50 paternal half-sib families (Figs. 3 and 4 and see Additional file 2).

With a clear population structure of paternal half-sib families in the validation population, we investigated how the conditional algorithm spreads core animals. In Fig. 4, we visualise UMAP for the validation population and mark chosen core animals. With just a few exceptions, the conditional algorithm spread the core animals across the validation population by selecting a core animal from each half-sib family. By comparison, the random construction would randomly spread core animals across the validation population, each time differently.

**Fig. 5:**
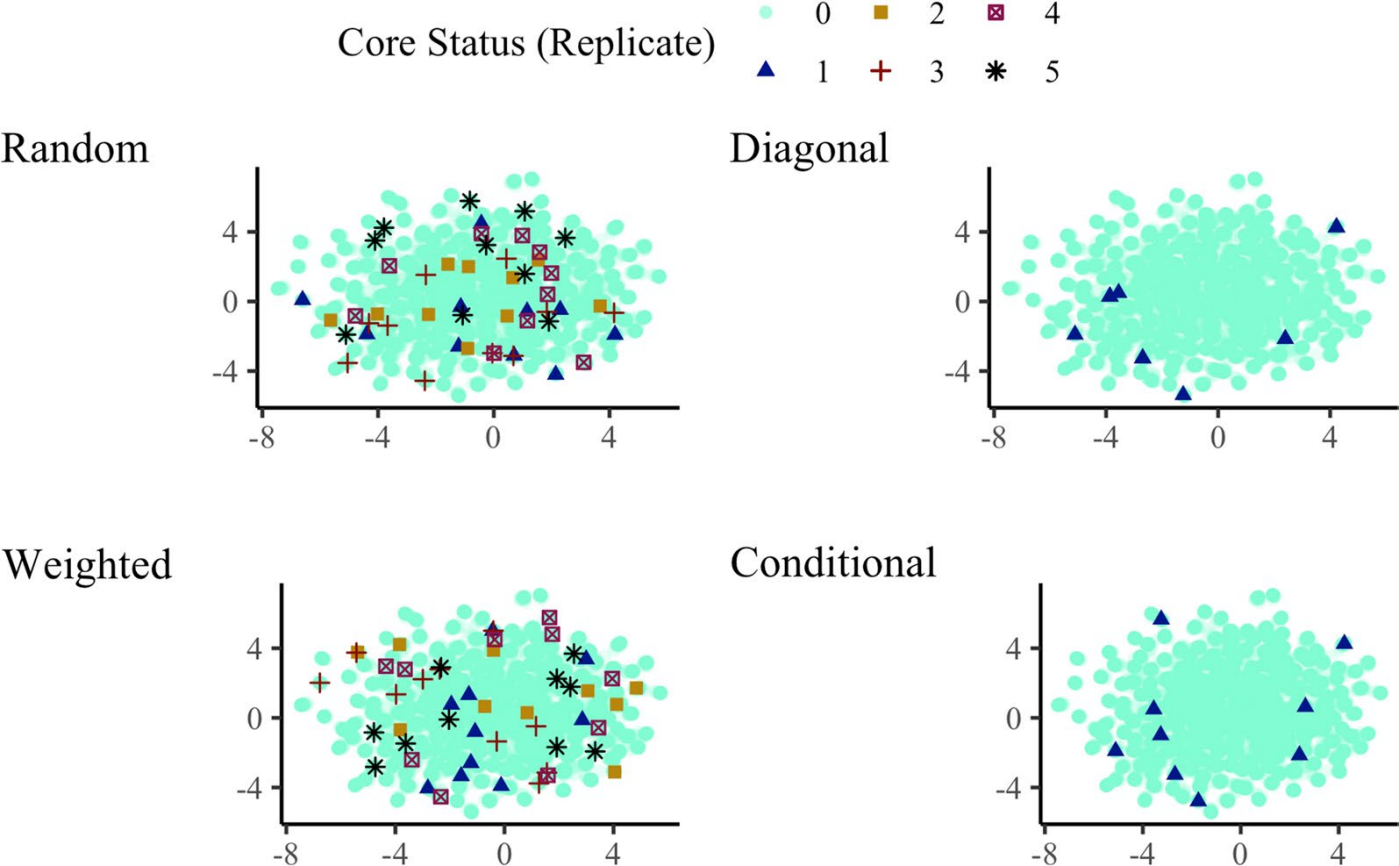
Spread of core animals with different core subset constructions in simulation. Ten core animals, from each of the four core subset constructions, are plotted on the Uniform Manifold Approximation and Projection (UMAP). Core animals were either selected at random (Random), based on the highest diagonal in $\mathbf {G}$ (Diagonal), a combination of Random and Diagonal (Weighted), or the conditional algorithm (Conditional). Random and Weighted show core animals from five samples (1–5), Diagonal and Conditional from one sample (1), while non-core animals are shown as zero (0)

Figure 5 shows UMAP for the 15,000 animals in simulation and 10 core animals from the four core subset constructions. Random constructions show core animals from five samples to indicate variability. This figure again demonstrates how the core animals are spread across a population and how these core animals differ between independent constructions. For example, no core animals overlapped across all five samples, and on average only $\sim$15% of core animals overlapped between pairs of replicates. By design, there is no such variability in the diagonal and conditional constructions—core animals are always the same for a given core subset size and a given set of genotyped animals. Note that some chosen core animals are not displayed since they have been placed outside the chosen axis limits.

### Real pig data

For the real pig dataset, the number of core animals was 8, 60, 184, 485, 1658, 2926, 5546, and 8348. These core subset sizes corresponded to the number of largest eigenvalues that captured 10, 30, 50, 70, 90, 95, 98, and 99% of the variation in $\mathbf {G}$. Note that there is a difference between the explained variation in $\mathbf {G}$ used to define the number of core animals and the “realised” variation in $\mathbf {G}$ explained by those core animals. The first definition quantifies the percentage of variation in $\mathbf {G}$ captured by a particular number of largest eigenvalues. The second definition quantifies how much variation in $\mathbf {G}$ is actually captured by a core subset with a particular number of animals. All the results presented here are based on the first definition. Alternatively, the realised variation can be obtained by dividing the trace of $\mathbf {G_{cc}}$ by the trace of $\mathbf {G}$. For smaller core sizes, there were no meaningful differences between the realised variation for the core subset constructions (see Additional file 3). For larger core sizes, the diagonal core subset construction captured 1 to $2\%$ more variation in $\mathbf {G}$ compared to the other methods, which is expected. The realised variation by the core subsets was much lower than the explained variation by the largest eigenvalues.

**Fig. 6:**
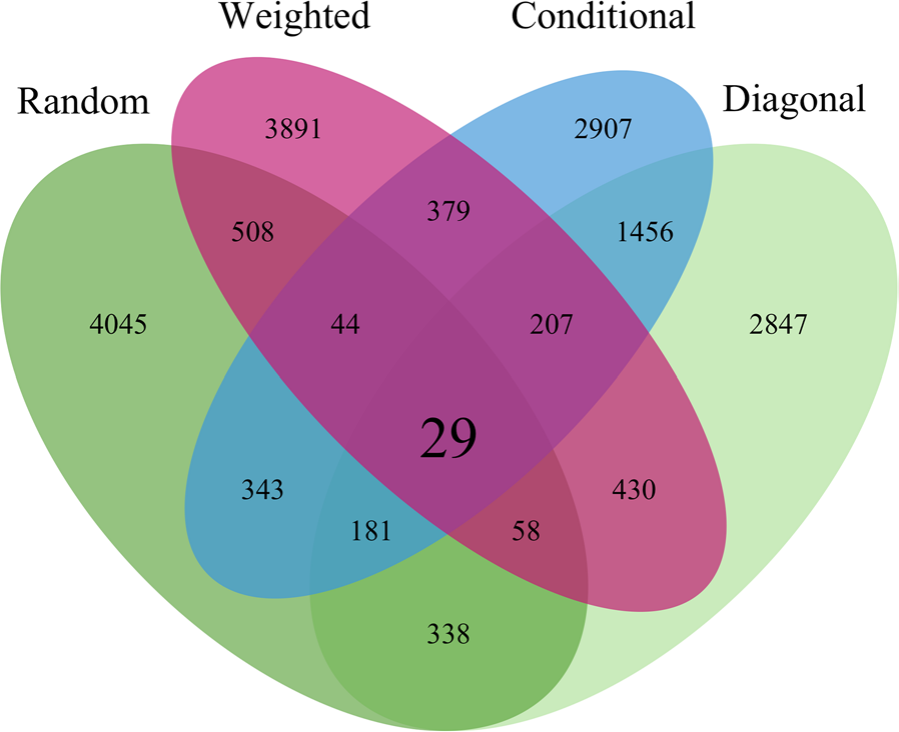
Venn diagram of core animals from the different core subset constructions in pigs. The Venn diagram shows overlap for 5546 core animals between the four core subset constructions. Core animals were selected at random (Random), based on the highest diagonal in $\mathbf {G}$ matrix (Diagonal), a combination of Random and Diagonal (Weighted), or the conditional algorithm (Conditional). For Random and Weighted only a single replicate is shown

The Venn diagram in Fig. 6 shows the number of shared core animals between the four core subset constructions. The total number of core animals is 5546, which corresponded to the number of largest eigenvalues that captured 98% of the variation in $\mathbf {G}$. Most core animals were shared between the diagonal and conditional constructions (34%, 1873/5546), while less than 1% (29/5546) were shared across all four core subset constructions. The random construction produced the most unique core subset, with 73% (4045/5546) of core animals not shared with any other construction. For the random and weighted constructions, the results were consistent across all replicates, but we show only a single replicate for clarity. Furthermore, the Venn diagram in Additional file 4 shows the number of shared core animals between five replicates of the random construction. In particular, there were no core animals shared across all five replicates, but at least 392 were shared between pairs of replicates. A similar result was observed for the simulated data (not shown).

**Fig. 7:**
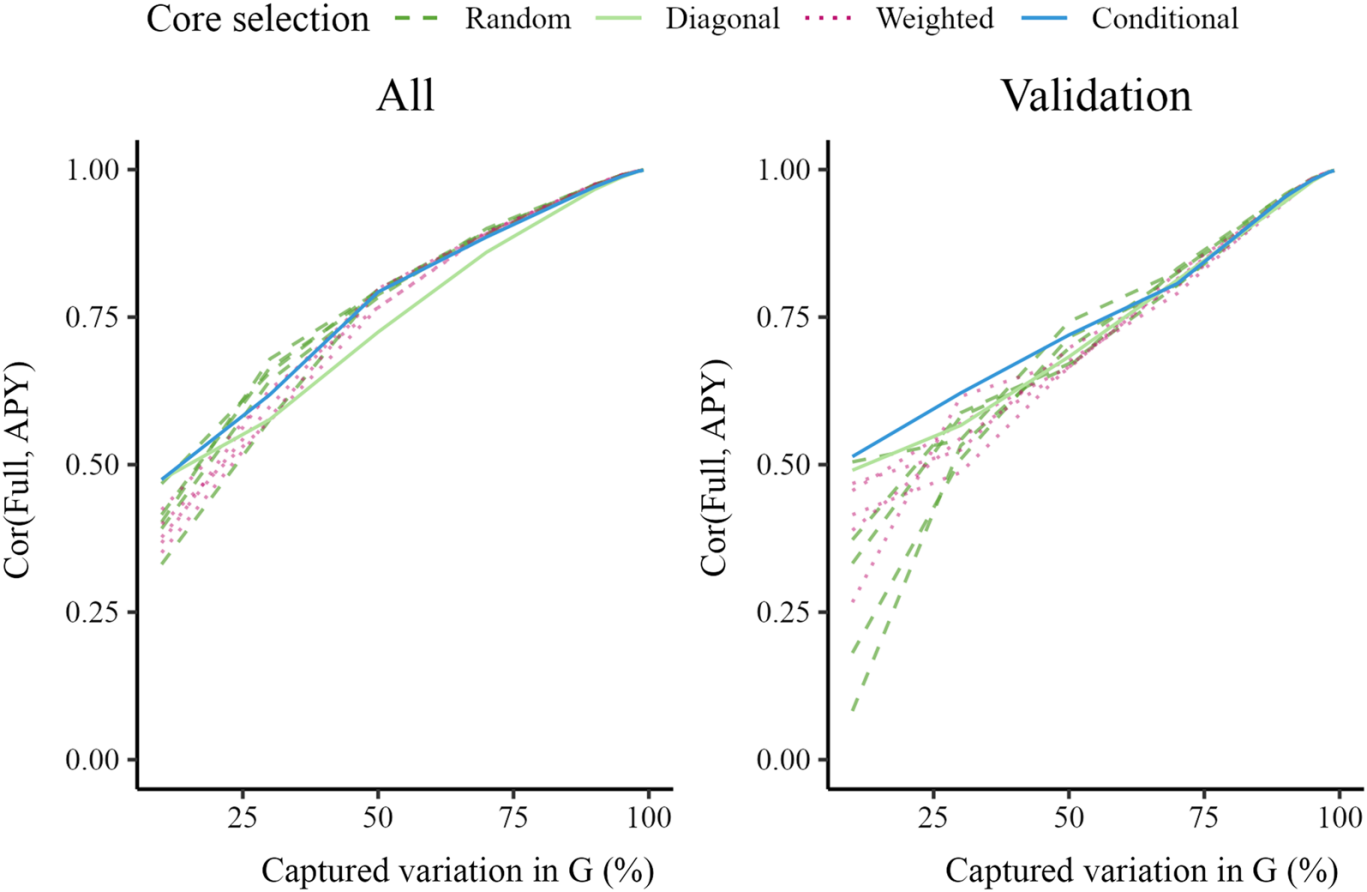
Correlations between $\mathbf {GEBV_{Full}}$ and $\mathbf {GEBV_{APY}}$ for all pigs and just validation pigs. Genomic estimated breeding values (GEBV) were based on the full inverse (Full) or the Algorithm for Proven and Young inverse (APY) of the genomic relationship matrix ($\mathbf {G}$). For APY, the core subset was constructed at random (Random), based on the highest diagonal in $\mathbf {G}$ (Diagonal), a combination of Random and Diagonal (Weighted), or the conditional algorithm (Conditional). Random and weighted constructions show five samples

Figure 7 shows that correlations between GEBV obtained with the full and APY inverse in real data were almost a linear function of the percentage of variation captured with the APY approximation. All core subset constructions performed similarly well when the core subset was large, with correlations greater than 0.99 when the number of core animals corresponded to the number of largest eigenvalues that captured 98% of the variation in $\mathbf {G}$. The difference between the four constructions was greater in the validation subset than in the whole genotyped population. The diagonal construction produced lower correlations than the other constructions for the full genotyped population, but not for the validation subset. There was a large degree of variability between the five replicates for the random and weighted constructions when the number of core animals was low, especially for the validation subset. The conditional construction often had the highest correlation, particularly in the validation subset, but not always.

**Fig. 8:**
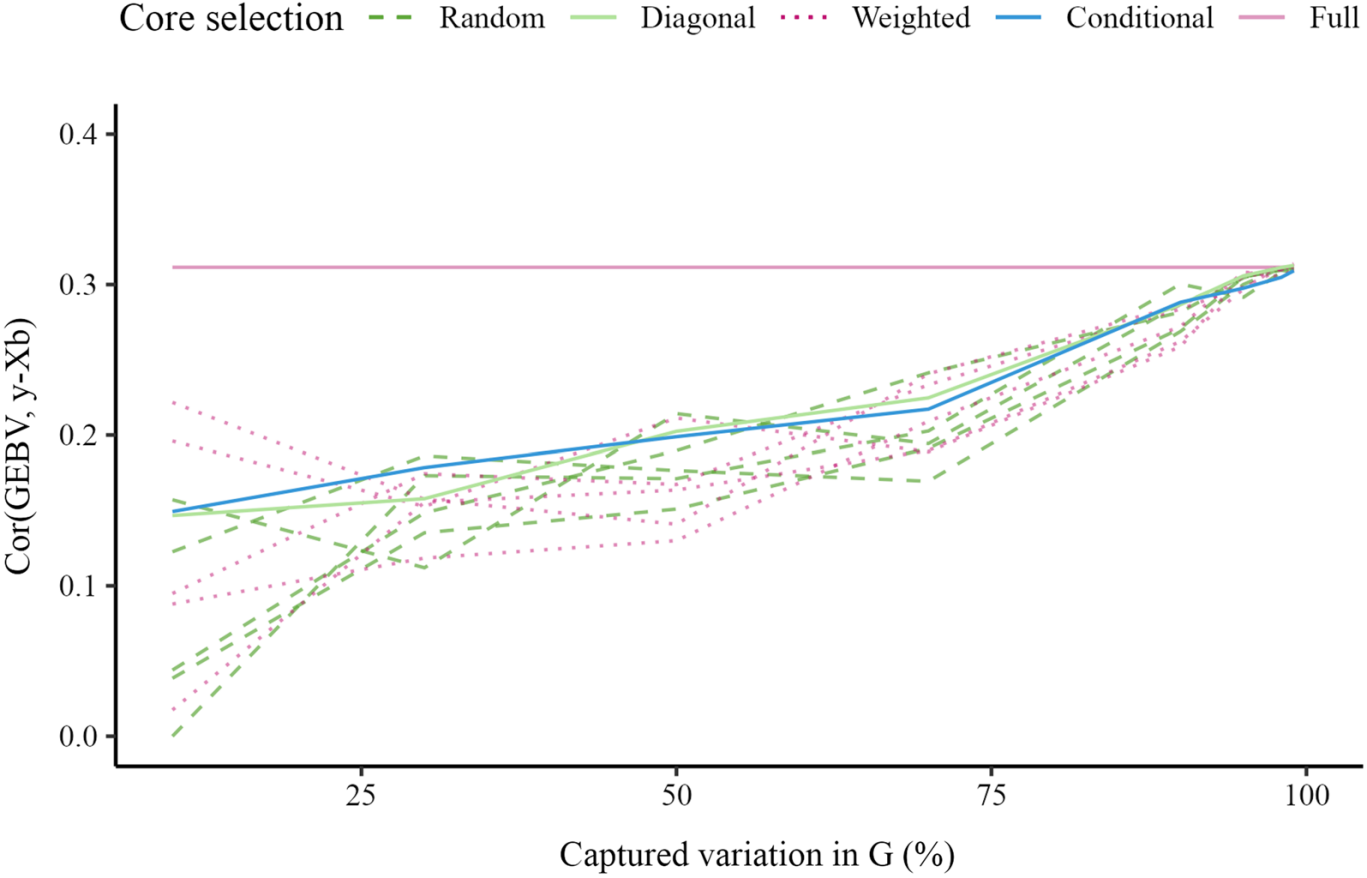
Predictive ability for the validation set in pigs. Accuracy is the correlation between genomic estimated breeding values (GEBV) and phenotypes adjusted for the fixed effects ($\mathbf {y-Xb}$). The GEBV were based on the full inverse (Full) or the Algorithm for Proven and Young (APY) inverse of the genomic relationship matrix ($\mathbf {G}$). For APY, the core subset was constructed at random (Random), based on the highest diagonal in $\mathbf {G}$ (Diagonal), a combination of Random and Diagonal (Weighted), or the conditional algorithm (Conditional). Random and weighted constructions show five samples

Figure 8 shows the accuracy of GEBV for validation animals relative to the percentage of variation captured in $\mathbf {G}$. For example, the accuracy of GEBV obtained with the full inverse was 0.31, and when the number of core animals corresponded to the number of largest eigenvalues that captured 90% of the variation in $\mathbf {G}$, the accuracy of the random construction was 0.28 (0.27 to 0.30), the diagonal construction was 0.29, the weighted construction was 0.27 (0.26 to 0.29) and the conditional construction was 0.29. Again, the conditional construction generally had the highest accuracy. The accuracy of the random construction was higher than that of the conditional construction when the number of core animals corresponded to the number of largest eigenvalues that captured more than 95% of the variation in $\mathbf {G}$, but note that the plotted difference is more pronounced by the rounding of accuracies to two decimal digits. The difference between the two constructions was always less than 0.005. The accuracy of the diagonal construction marginally surpassed the full inverse when the number of core animals corresponded to the number of largest eigenvalues that captured 99% of the variation in $\mathbf {G}$. In this sense, the conditional construction achieved the same satisfactory accuracy as the random construction when the number of core animals was large, but also achieved notable improvements over the random construction when the number of core animals was smaller. This improvement can be demonstrated by comparing the accuracy from the conditional construction with the large variability in accuracy from the random and weighted constructions for a small number of core animals.

In addition to accuracy, we also assessed the dispersion bias for validation animals as shown in Additional file 5. In terms of dispersion bias (regression coefficients), there were no differences between the core subset constructions. In general, all four core subset constructions, as well as the full inverse, exhibited similar inflation (overestimation) of the GEBV. In addition, we analysed the number of rounds to achieve convergence (see Additional file 6). The random and conditional core subset constructions needed fewer rounds to achieve convergence compared to the diagonal core subset construction. In general, we did not observe any major differences in the convergence patterns between different core subset constructions.

We visualised the structure of the pig population with PCA and UMAP as shown in Additional file 7. PCA showed clear clusters of crossbreed animals (F1, BC1, and BC2) and purebred animals (L1). As expected, the backcross animals were positioned between the purebred and F1 animals. UMAP also showed clusters of animals, but while PCA showed L1 as a homogeneous population in first two dimensions, UMAP revealed additional structure within the L1 animals in the first two dimensions. Closer inspection of the data revealed that this additional structure corresponds to a time trend within a breeding programme (not shown).

**Fig. 9:**
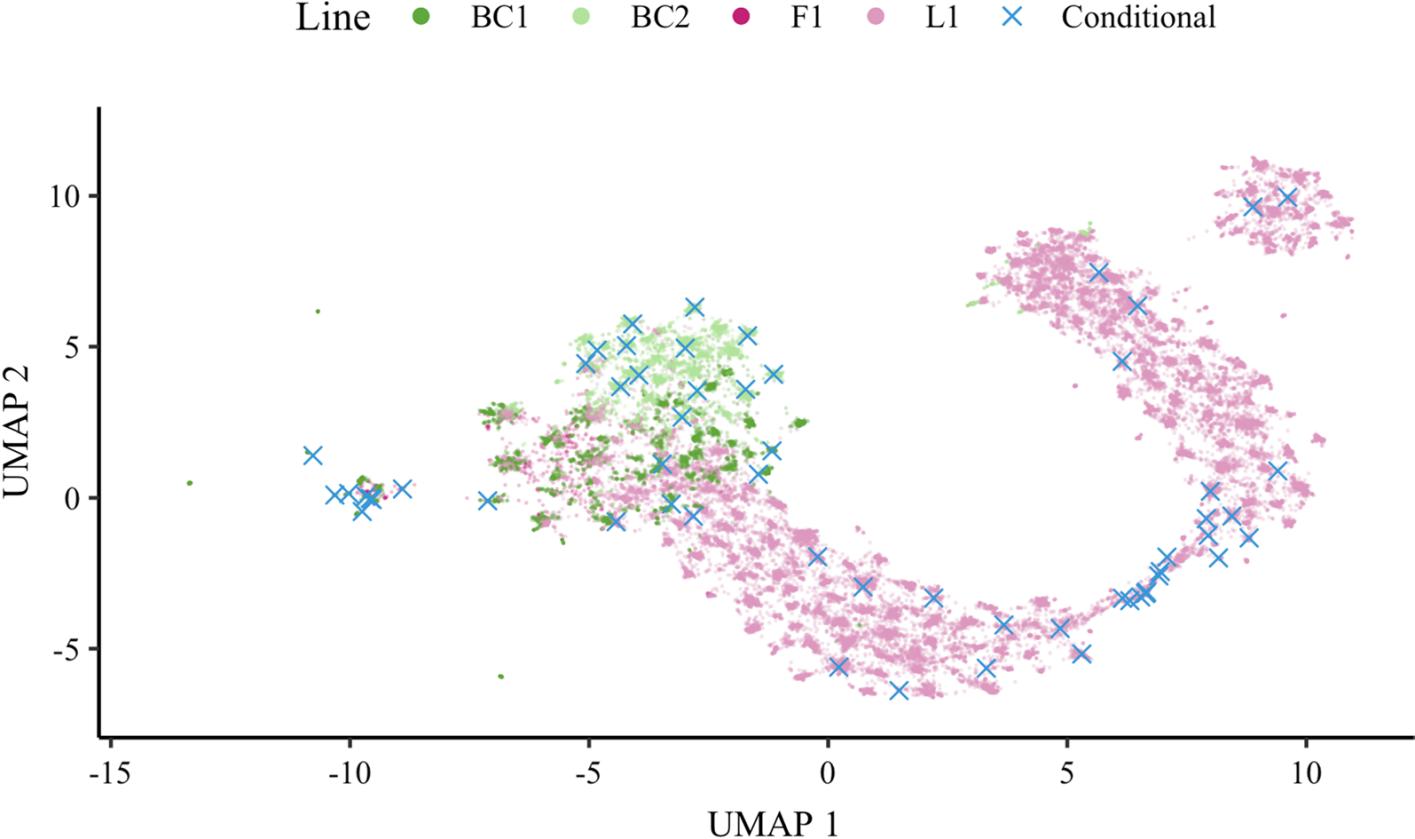
Visualisation of UMAP for genotyped pigs and an example of the optimised core subset for 60 pigs. Animals chosen by the conditional algorithm (Conditional) are plotted on the Uniform Manifold Approximation and Projection (UMAP). Colours represent purebred (L1), crossbred (F1), and backcross (BC1, BC2) pigs

Figure 9 illustrates how the conditional algorithm spreads animals, with an example of 60 core animals to facilitate visualisation. In this example, the conditional algorithm chose 14 BC1, 13 BC2, 1 F1, and 32 L1 animals. For comparison, one random sample chose 11 BC1, 5 BC2, 2 F1, and 42 L1 animals.

## Discussion

The results show that core subset construction can be optimised and that such optimisation delivers accurate and stable GEBV. Our results raise five points for discussion: (i) the overall performance of APY and the need for optimising the core subset; (ii) towards optimal core subset construction; (iii) expanding the core subset with new genotype data; (iv) limitations; and (v) other opportunities.

### The overall performance of APY and the need for optimising the core subset

Our results confirm that the size of the core subset is critical in APY and that robust results can be obtained with a sufficiently large core subset. The results also show that given the size of the core subset, the proposed conditional algorithm achieves an optimal and repeatable spread of core animals across the domain of genotyped animals. We observed a similar increase in accuracy when increasing the core subset size for the simulated and real datasets. However, there was more variability in accuracy with the random construction for the real dataset than for the simulated dataset. Furthermore, the difference between core subset constructions was more apparent in the real dataset, especially with a small core subset. This was most likely because the real dataset has a more complex structure than the simulated dataset, which again justifies the need for optimisation. Although the relationship between the size of the core subset and the accuracy of GEBV is well described [19, 20, 22, 37], construction of the core subset is somewhat neglected, with the random construction being the most common. The proposed conditional construction addresses this situation. While the conditional construction requires more computation time than the random construction, this additional time could be justified in several cases, specifically when the genotyped population has a complex structure, such as for multiple breeds or crossbred animals.

Ostersen et al. [15] and Bradford et al. [20] provided early indications that the choice of animals for the core subset is important in specific cases. For example, in the case where genotyped animals had an incomplete pedigree (with a variable number of generations), spreading the core animals across multiple generations maximised accuracy [15, 20]. Our results show that complex population structures, like for multiple breeds or crossbred animals, necessitate some optimisation of the core subset. Mäntysaari et al. [7] mentioned this issue in their genetic evaluation involving 41 breeds, but did not provide a recommendation on how to optimise the core subset. Vandenplas et al. [38] simulated a three-way crossbreeding program, and observed that the best accuracy was achieved when the core animals were randomly chosen within each breed and crossbred population. This simulation was later corroborated with real data [16]. Vandenplas et al. [38] also suggested that statistically or numerically more “stable” core subsets might be needed for populations with a complex population structure, even if this increases computing cost. Their approach with the QR decomposition of the SNP genotype matrix chose a core subset that improved convergence, but it did not improve accuracy. Nilforooshan and Lee [17] used the diagonal construction based on $\mathbf {G}$ as well as based on $\mathbf {A}$. They found that the diagonal construction based on $\mathbf {G}$ gave a similar correlation between GEBV from the full inverse and APY inverse, when the core subset was sufficiently large, but not for smaller core subsets. We observed similar results with the real dataset. Recently, Cesarani et al. [39] used APY in a large-scale genomic evaluation including five breeds. They observed changes in the prediction accuracy with different choices of core animals. They concluded that the random choice of core animals impacts prediction accuracy because breeds with less genotyped animals are not well represented. To address this undersampling, they proposed increasing the core subset size and balancing the core subset size per breed. In their case, this leads to the core (total number of genotyped animals) including 15k (3.4M) Holstein, 15k (400k) Jersey, 5k (9k) Ayshire, 5k (47k) Brown Swiss, and 5k (5k) Guernsey animals. Nevertheless their within-breed core subsets were still random. The conditional algorithm could optimise such a core subset because it works with SNP genotypes that capture both between- and within-breed variation.

### Towards optimal core subset construction

To our knowledge, there have been no previous attempts to statistically optimise the APY core subset, except from the perspective of ensuring better convergence [38]. All other attempts largely modified the random approach by adding additional criteria, like spreading random choice within generations, groups of animals (e.g., bulls, cows, most inbred, popularity, etc.), or breeds [2, 15, 17, 20, 22, 38]. Our conditional core subset construction follows ideas from sequential sampling, e.g. [29, 30], in which sampling is based on the variance of SNP genotypes for each individual (diagonal of the $\mathbf {G}$) conditional on the current core subset. This results in a subset of core animals which are spread across the domain of genotyped animals and equivalently across the domain of collected SNP genotype data.

UMAP was used to gain insight into the spread of core animals across each population. On the one hand, for the simulated dataset, UMAP clearly distinguished each generation and each half-sib family (within generation). It also revealed that the conditional construction commonly chose one core animal per half-sib family. On the other hand, the random construction samples core animals at random and can sub-optimally cover the domain of genotyped animals. For the real dataset, UMAP clearly revealed the population structure across breeds and time as well as between family variation. Visualisation of the core animals chosen by the conditional construction showed how these animals were optimally spread across the domain of genotyped animals with extensive population structure. Of note, UMAP on the real data produced some very distant outliers, which we have omitted to improve visualisation (see Additional file 8).

The results show how the conditional construction mitigates fluctuations in accuracy present with the random construction on smaller core subsets. Venn diagrams (Fig. 6 and Additional file 4) showed striking differences between different core subset constructions, but even more importantly between replicates of the random construction. For example, in our simulated and real datasets, the random construction had no overlap in core animals across five replicates. As expected, fluctuations in accuracy were larger with smaller core subsets and smaller with larger core subsets. While large core subsets are the norm and easy to accommodate on modern computing infrastructure, the conditional algorithm can be useful to reduce even the smallest fluctuations in accuracy. For example, in the simulation we saw variation in selected sires even when the core subset captured most of the variation in $\mathbf {G}$.

It is important to point out that the conditional algorithm does not always provide the globally optimal core subset. This was clearly seen in the results, where sometimes other core subset constructions had marginally higher accuracy. The conditional algorithm starts with one core animal and then sequentially grows the core subset in a repeatable manner by choosing an animal whose SNP genotypes are least captured by the current core subset (that is, the animal has the largest conditional variance). However, a different start can produce a different core subset. For consistency, we always started with the animal with the largest diagonal value in $\mathbf {G}$, hence the algorithm started on the farthest edge of the domain of genotyped animals. Alternatively, we could have started with the animal closest to the centroid of SNP genotypes, hence the algorithm would have started in the centre of the domain. While achieving global optimality is difficult, we attempted to improve the optimisation by sequentially choosing core animals that would maximise the captured variation across the domain (that is, minimising the conditional variance in the next iteration). However, such an algorithm was impractically slow. Further work is required to develop more optimal algorithms for core subset construction.

### Expanding the core subset with new genotype data

Optimising expansion of the APY core subset with the arrival of new genotype data has not been addressed in the literature although several empirical studies have been done [40–42]. When new genotype data arrive, we can either construct a new core subset or expand the existing core subset. The proposed conditional algorithm can expand an existing core subset because it works with the original or a conditional SNP genotype matrix. To expand the core subset with new genotype data we have to (1) condition the SNP genotype matrix of new animals on the old core subset, (2) combine the conditional SNP genotype matrices of old core and new animals, and (3) run the conditional algorithm on the the combined conditional SNP genotype matrix. Conditioning the SNP genotype matrix of new animals on the old core subset can be done efficiently with Algorithm 3, where we combine the SNP genotype matrices of core and new animals and sequentially condition the combined SNP genotype matrix on each core animal (line 5). We provide an implementation of this algorithm in our code (https://github.com/HighlanderLab/ipocrnic_OptimisedCore4APY and https://doi.org/10.5281/zenodo.7181323). We have found that the same core subset is obtained by either running a combined optimisation (Algorithm 2 on the combined SNP genotype matrix) or expanding the core subset (Algorithm 3).
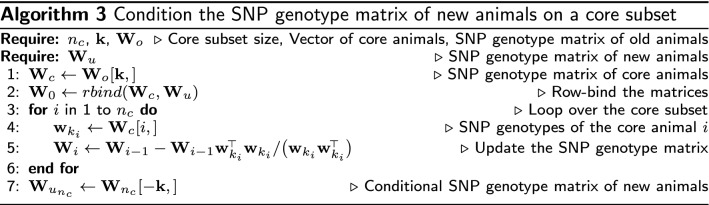


The described approach to expand the core subset assumes that the same allele frequencies are used in centering the SNP genotype matrix of old and new animals. Ideally, we would use the combined allele frequencies of old and new animals. However, the old core subset was selected based on old allele frequencies. Hence, the same allele frequencies must be used for new animals too. This is not ideal since some routine genetic evaluations continually update allele frequencies, although changes between genetic evaluations are likely small, even in breeding programmes. Some genetic evaluations use base population allele frequencies, which are expected to change even less.

Garcia et al. [41] tested the stability of APY-based indirect predictions when large numbers of genotyped animals are added to the dataset. They concluded that provided the number of core animals is sufficiently large, APY-based indirect predictions are stable, irrespective of the core subset definition and the number of new genotypes. Similarly, Hidalgo et al. [42] examined the impact of adding new data with or without updating the core subset. They found that only slight changes in GEBV occurred when the core was updated, compared to using a fixed core for the same period of time. Therefore, in line with the recommendations in Misztal et al. [21], Hidalgo et al. [42] recommend using the same core subset for a longer period of time (for example, 1 year), and update it when a significant amount of new data is generated. This update can be optimised with the conditional algorithm proposed in this paper.

### Limitations

The main limitation of the conditional core subset construction is that it is more time consuming compared to the random construction. We see two options here. The first option is to work with a reduced rank SNP genotype matrix. In terms of the simulated data set, we showed that iterating over the reduced rank SNP genotype matrix halved the computation time with no loss in accuracy, although the core subset was somewhat different. Therefore, while iterating over a reduced rank SNP genotype matrix saves computation time, it potentially adds another complexity, particularly when we also have to consider expansion with new data. The second option is to select groups of animals using the “selector” vector $\mathbf {e}$ instead of selecting just one animal at a time. This change will likely produce a sub-optimal result and more research is needed on balancing computation time versus accuracy and stability of the APY approximation.

### Other opportunities

This study provides opportunities for further research in relation to at least four other study areas. First, the idea of using the conditional variance for choosing core animals in APY is identical to choosing animals for high-density genotyping or sequencing given a pedigree or low-density genomic relationship matrix [43]. Second, choosing core individuals that capture most of the genetic diversity is relevant also to designing and managing genebanks [44, 45]. Third, the conditional algorithm could be relevant to choosing a diverse subset for phenotyping where phenotyping is limited due to costs or other constraints [46, 47]. Fourth, inducing sparsity in dense inverse covariance (precision) matrices is an important topic in all areas that use Gaussian processes [48, 49]. Recently, Nearest Neighbour Gaussian Process (NNGP) has gained popularity for large-scale applications in spatial statistics [50]. In genetics, NNGP could define a core subset for each animal, which is similar to *Application 2* of Faux et al. [51] and ancestral regression of Cantet et al. [52] (where the core subset represents parents and grand-parents). Extension of these concepts could further optimise APY for multi-breed applications. For example, linking the non-core animals of one breed to the core animals of that breed, or to limit linking across too many generations in order to limit the contribution of older animals to recent generations.

## Conclusions

In summary, we have confirmed that the accuracy of genomic evaluation with APY depends on the size of the core subset. For a given size of the core subset we can optimise the core subset with the proposed conditional algorithm. This algorithm achieves an optimal and repeatable spread of core animals across the domain of genotyped animals.

## Supplementary Information

**Additional file 1.** Accuracy of the conditional core selection approach in simulation with rank reduction. Here we show accuracy as the correlation between genomic estimated breeding values (GEBV) and true breeding values in simulation. For the Algorithm for Proven and Young (APY), the core subset was optimised on either the full or rank reduced matrix **W**.

**Additional file 2.** Visualisation of UMAP over generations in simulation. Here we visualise Uniform Manifold Approximation and Projection (UMAP) for genotyped animals in each of the five generations (16, 17, 18, 19, and 20) in simulation.

**Additional file 3.** Percentage of realised variation explained in **G** by each core subset in pigs.

**Additional file 4.** Venn diagram of core animals from five random samples in pigs.The Venn diagram shows overlap for 5546 core animals between five (1–5) random samples.

**Additional file 5.** Bias for validation pigs as the regression coefficient from regression of their phenotypes (adjusted for the fixed effects) on their GEBV.

**Additional file 6.** Number of preconditioned conjugate gradient rounds (PCG) to convergence in pigs. PCG convergence criterion was set to 10^−12^ and was run using the BLUP90IOD2 software executed in The University of Edinburgh High-Performance Computing environment.

**Additional file 7.** Visualisation of PCA and UMAP for genotyped pigs. Projection of genomic relationships into first two dimensions was done with Principal Components Analysis (PCA) or with Uniform Manifold Approximation and Projection (UMAP). The percentage of variation captured by each principal component is shown in parentheses. Colours represent purebred (L1), crossbred (F1), and backcross (BC1, BC2) pigs.

**Additional file 8.** Visualisation of UMAP before and after cleaning the pig data. Here we visualise Uniform Manifold Approximation and Projection (UMAP) for genotyped purebred (L1), crossbred (F1), and backcross (BC1, BC2) pigs, before (a) and after (b) removing 378 (< 1%) pigs from the final UMAP plot for the sake of clarity.

## Data Availability

R code for simulation and core subset construction are publicly available at https://github.com/HighlanderLab/ipocrnic_OptimisedCore4APY and https://doi.org/10.5281/zenodo.7181323. The real pig data analysed in this study is owned by Genus PIC and is not publicly available.
