# Supplementary material for: Optimisation of the core subset for the APY approximation of genomic relationships
Source: Genet Sel Evol. 2022 Nov 22;54:76. doi: 10.1186/s12711-022-00767-x (PMC9682752; doi:10.1186/s12711-022-00767-x)
Supplement: Supplementary file 1 — Additional file 1. Accuracy of the conditional core selection approach in simulation with rank reduction. Here we show accuracy as the correlation between genomic estimated breeding values (GEBV) and true breeding values in simulation. For the Algorithm for Proven and Young (APY), the core subset was optimised on either the full or rank reduced matrix W. [file 12711_2022_767_MOESM1_ESM.docx]

**Additional File 1 (Table) - Accuracy of conditional core selection approach in simulation with rank reduction**. Accuracy is the correlation between genomic estimates of breeding values (GEBV) and true breeding values in simulation. For the Algorithm for Proven and Young (APY), core subset was optimised on either full or rank reduced **W** matrix.

| Number of core animals | Percentage of variation explained in G^1^ | Accuracy with full W^2^ | Accuracy with rank reduced W |
| --- | --- | --- | --- |
| 10 | 10 | 0.13 | 0.13 |
| 50 | 30 | 0.37 | 0.36 |
| 135 | 50 | 0.55 | 0.53 |
| 326 | 70 | 0.64 | 0.66 |
| 968 | 90 | 0.72 | 0.73 |
| 1516 | 95 | 0.74 | 0.74 |
| 2386 | 98 | 0.74 | 0.75 |

^1^**G** is the genomic relationship matrix

^2^**W** is the centred matrix of SNP genotypes
