# Supplementary figures and images for: Optimisation of the core subset for the APY approximation of genomic relationships
Source: Genet Sel Evol. 2022 Nov 22;54:76. doi: 10.1186/s12711-022-00767-x (PMC9682752; doi:10.1186/s12711-022-00767-x)

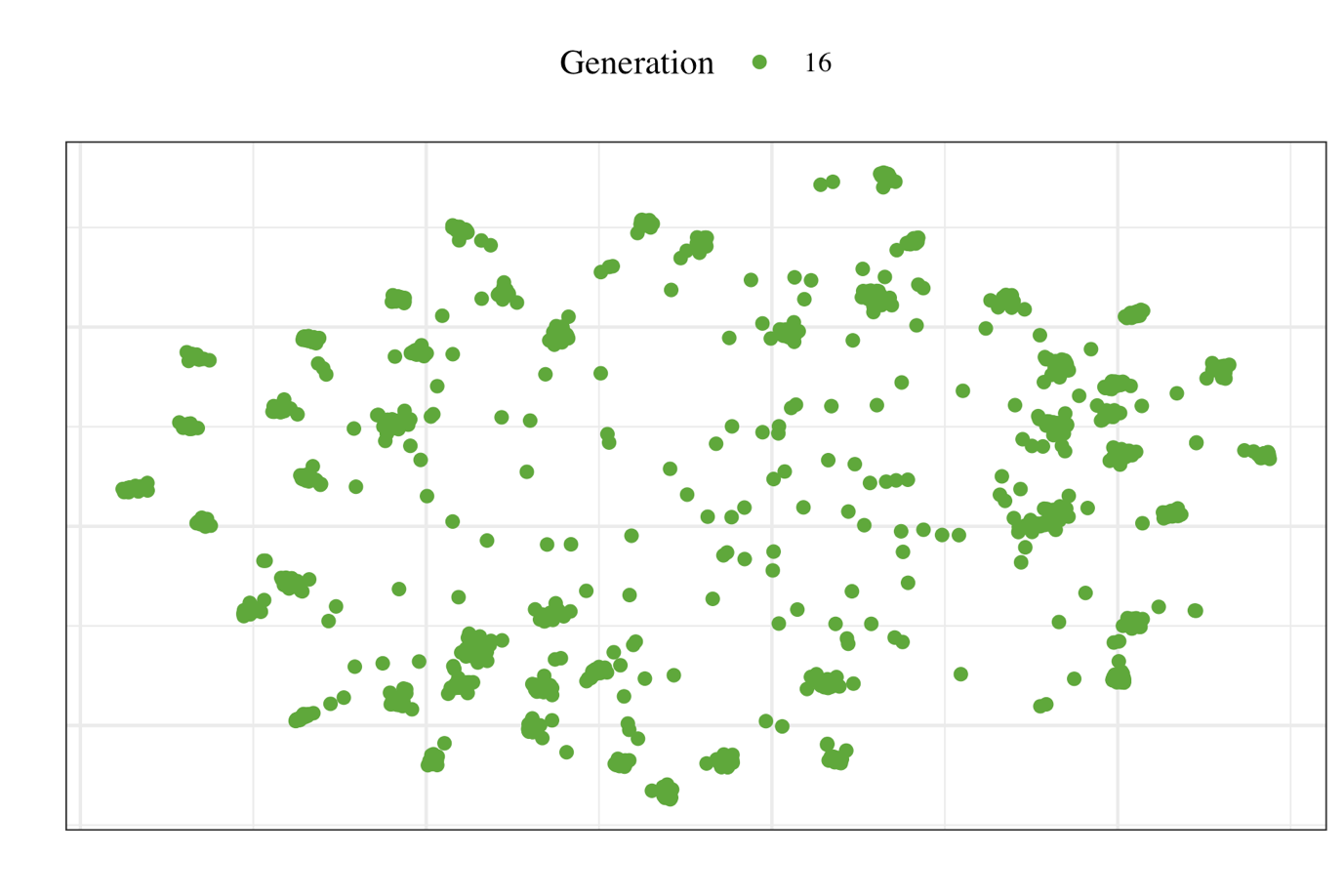


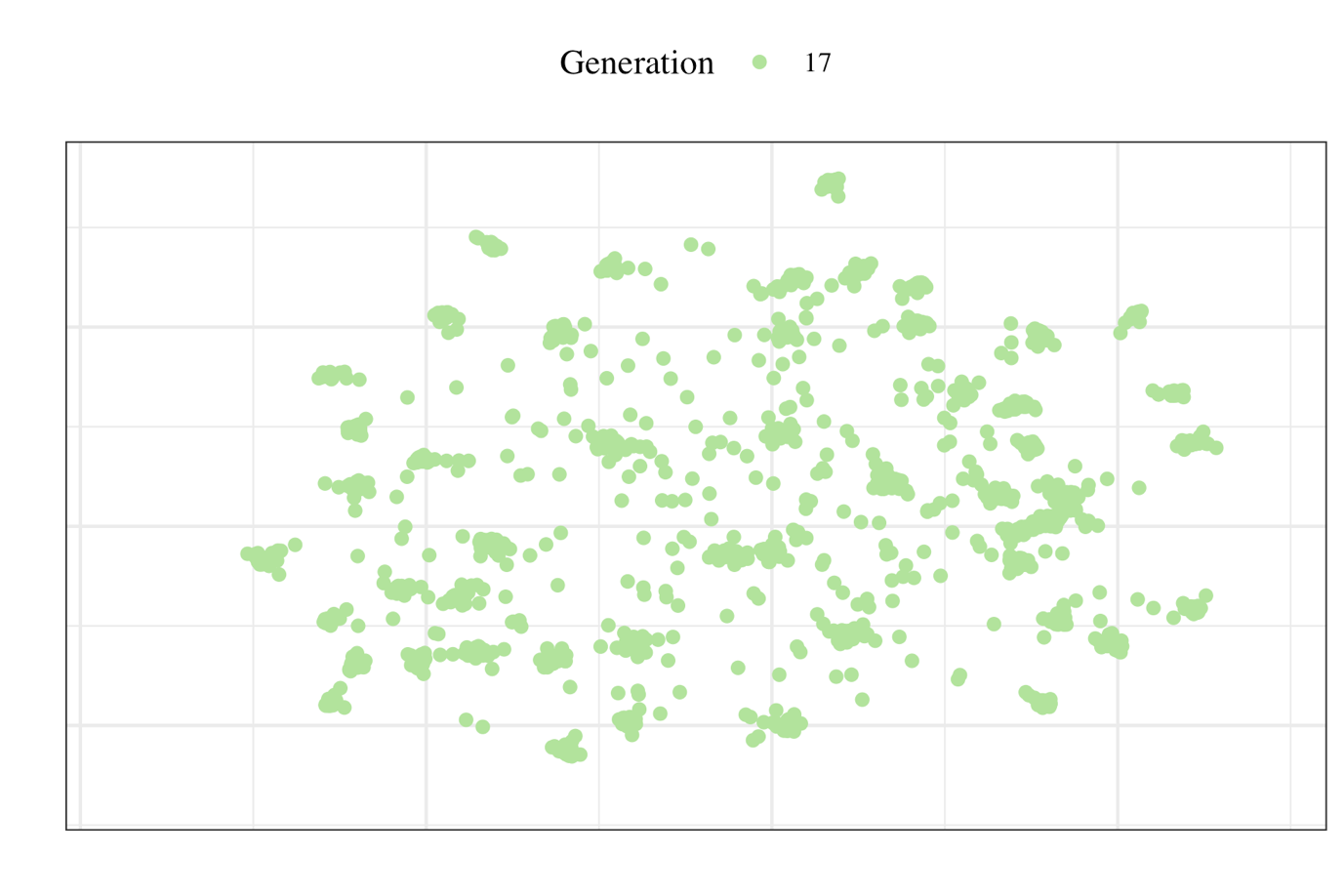


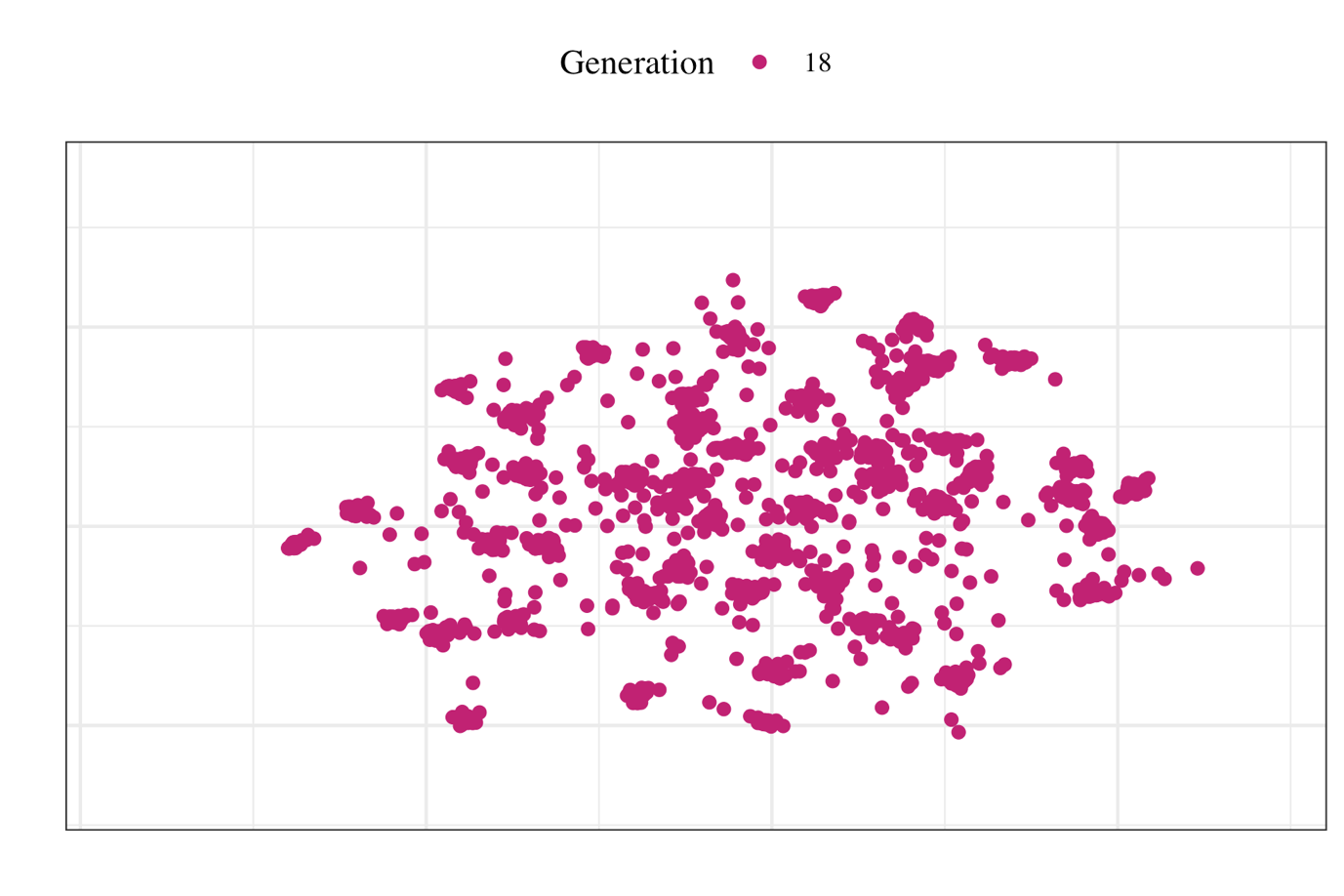


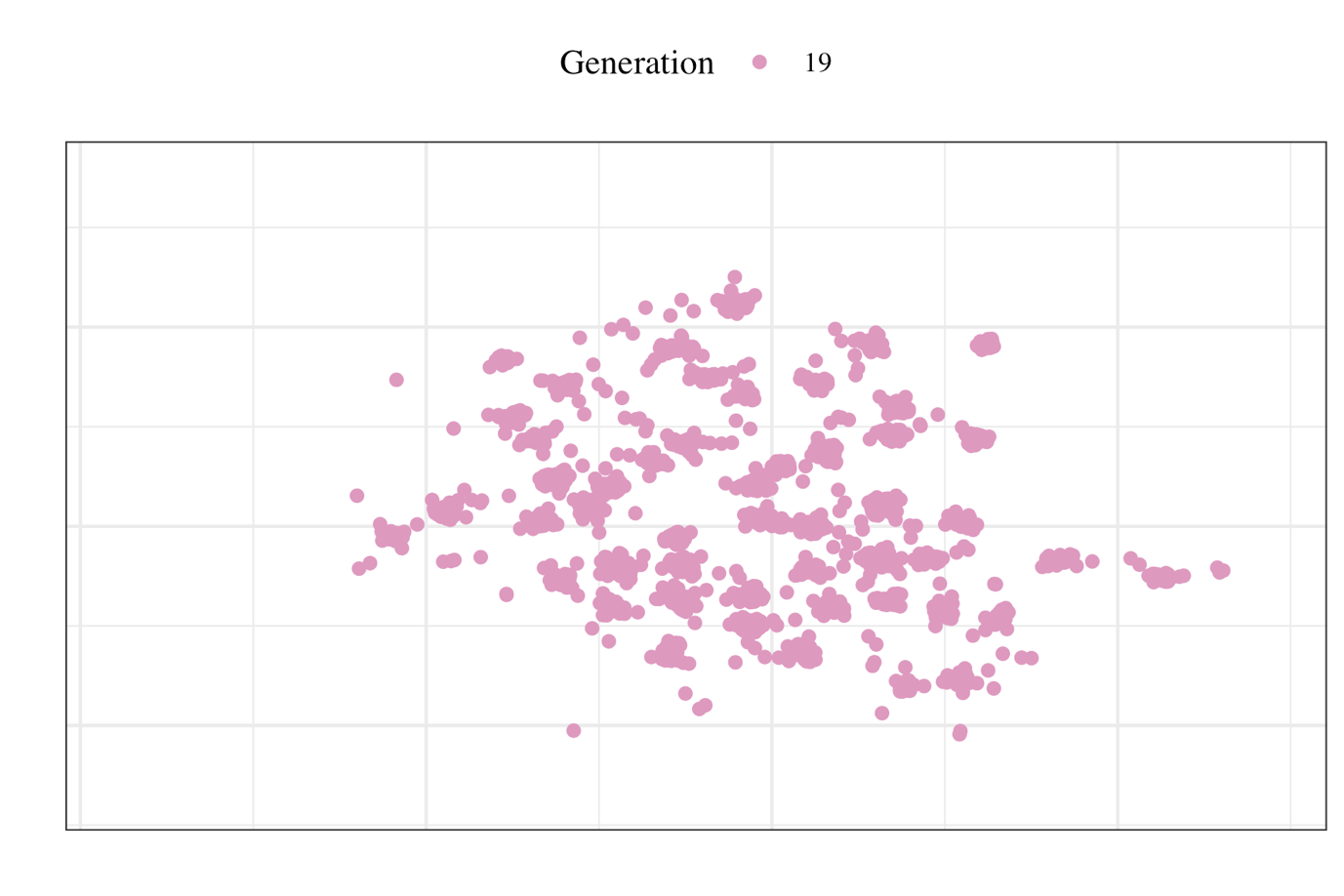


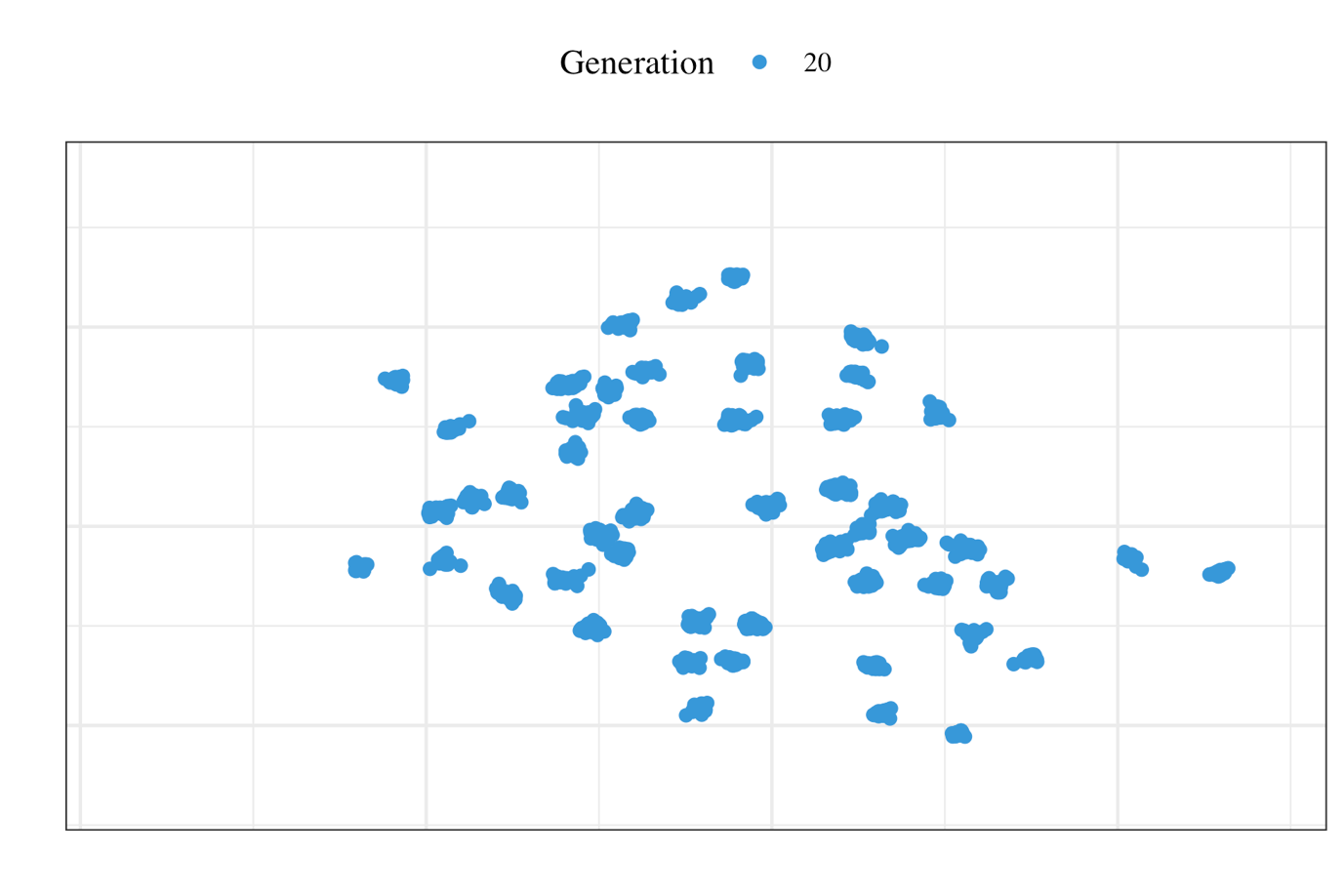

Supplement: Supplementary file 2 — Additional file 2. Visualisation of UMAP over generations in simulation. Here we visualise Uniform Manifold Approximation and Projection (UMAP) for genotyped animals in each of the five generations (16, 17, 18, 19, and 20) in simulation. [file 12711_2022_767_MOESM2_ESM.docx]

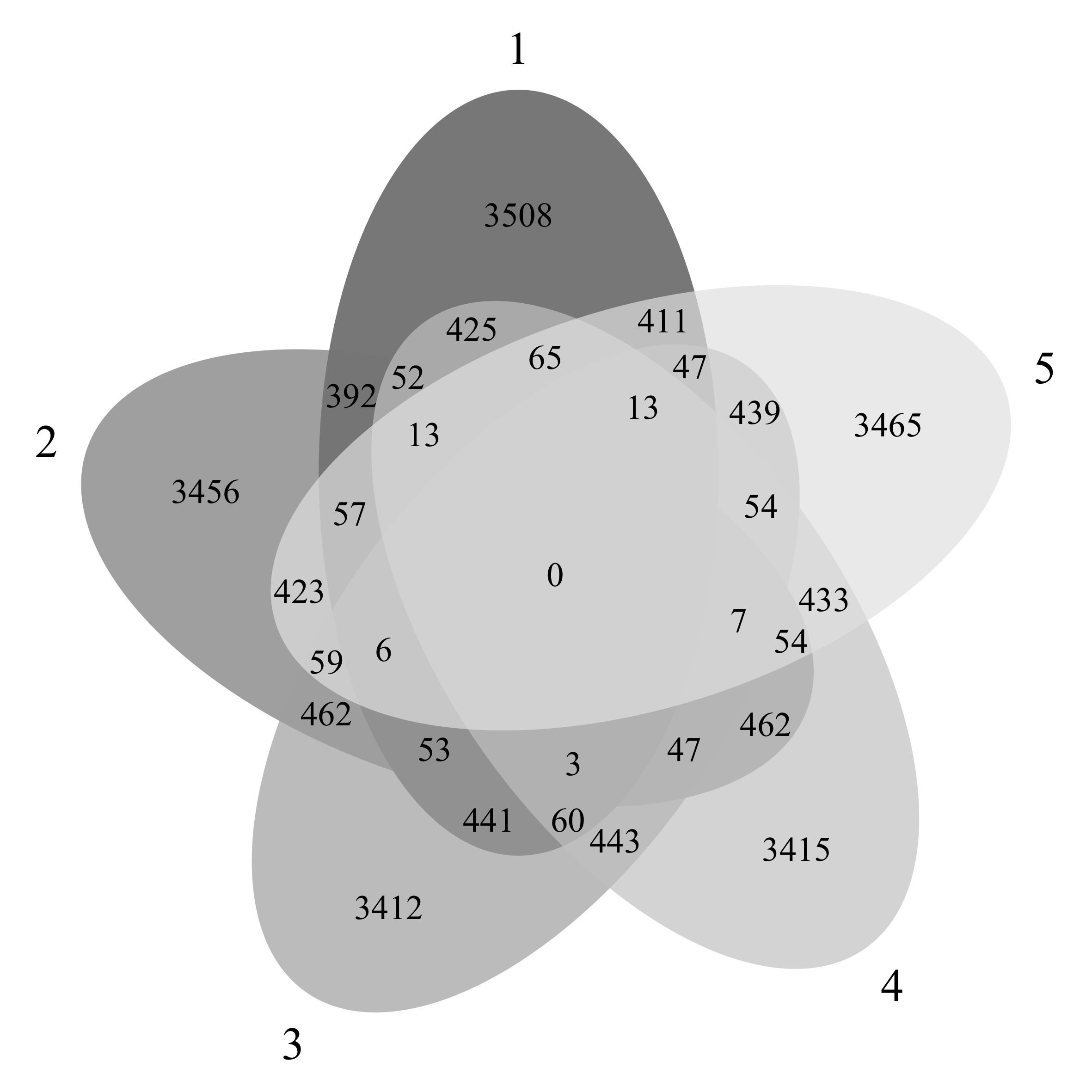

Supplement: Supplementary file 4 — Additional file 4. Venn diagram of core animals from five random samples in pigs.The Venn diagram shows overlap for 5546 core animals between five (1–5) random samples. [file 12711_2022_767_MOESM4_ESM.png]

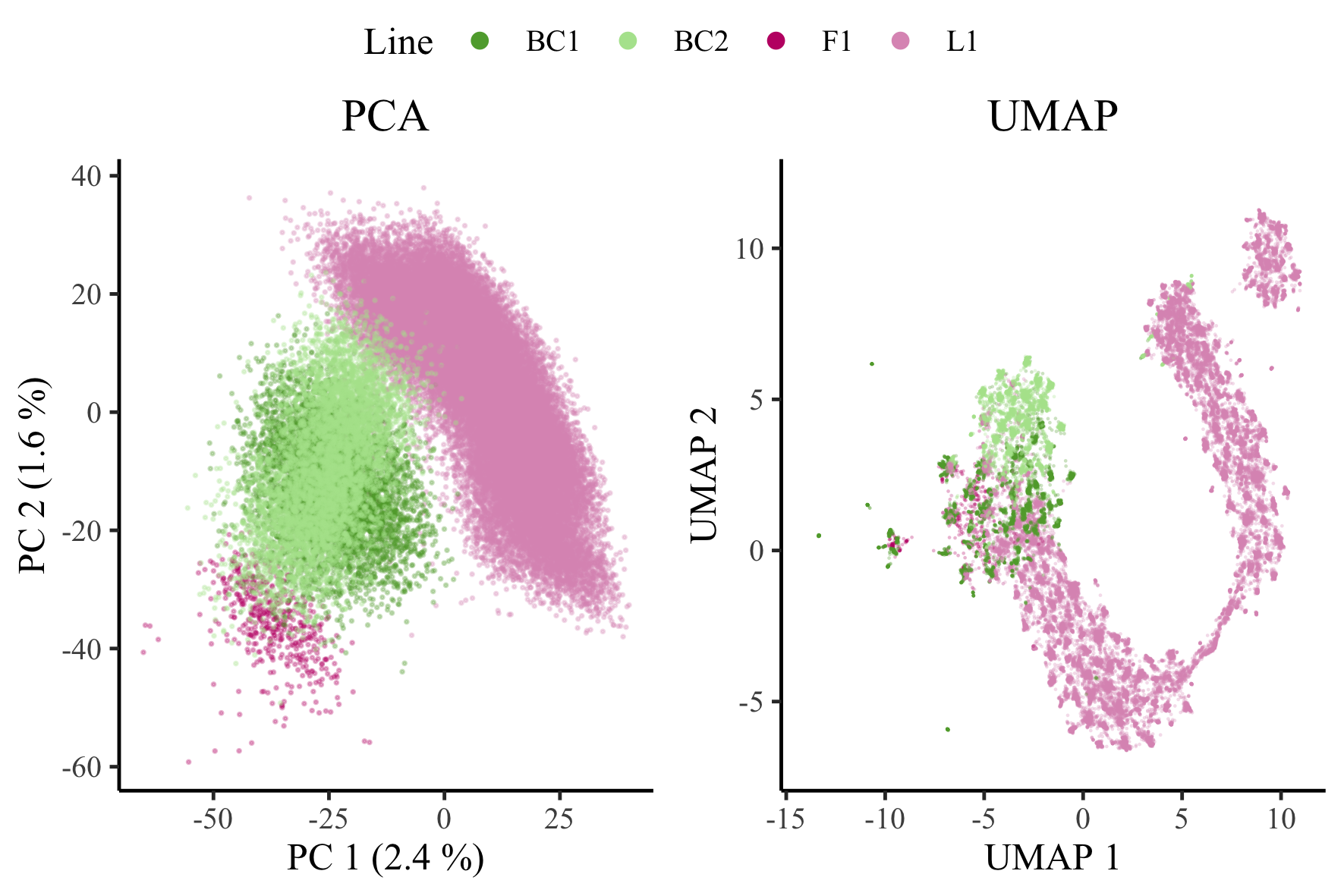

Supplement: Supplementary file 7 — Additional file 7. Visualisation of PCA and UMAP for genotyped pigs. Projection of genomic relationships into first two dimensions was done with Principal Components Analysis (PCA) or with Uniform Manifold Approximation and Projection (UMAP). The percentage of variation captured by each principal component is shown in parentheses. Colours represent purebred (L1), crossbred (F1), and backcross (BC1, BC2) pigs. [file 12711_2022_767_MOESM7_ESM.png]

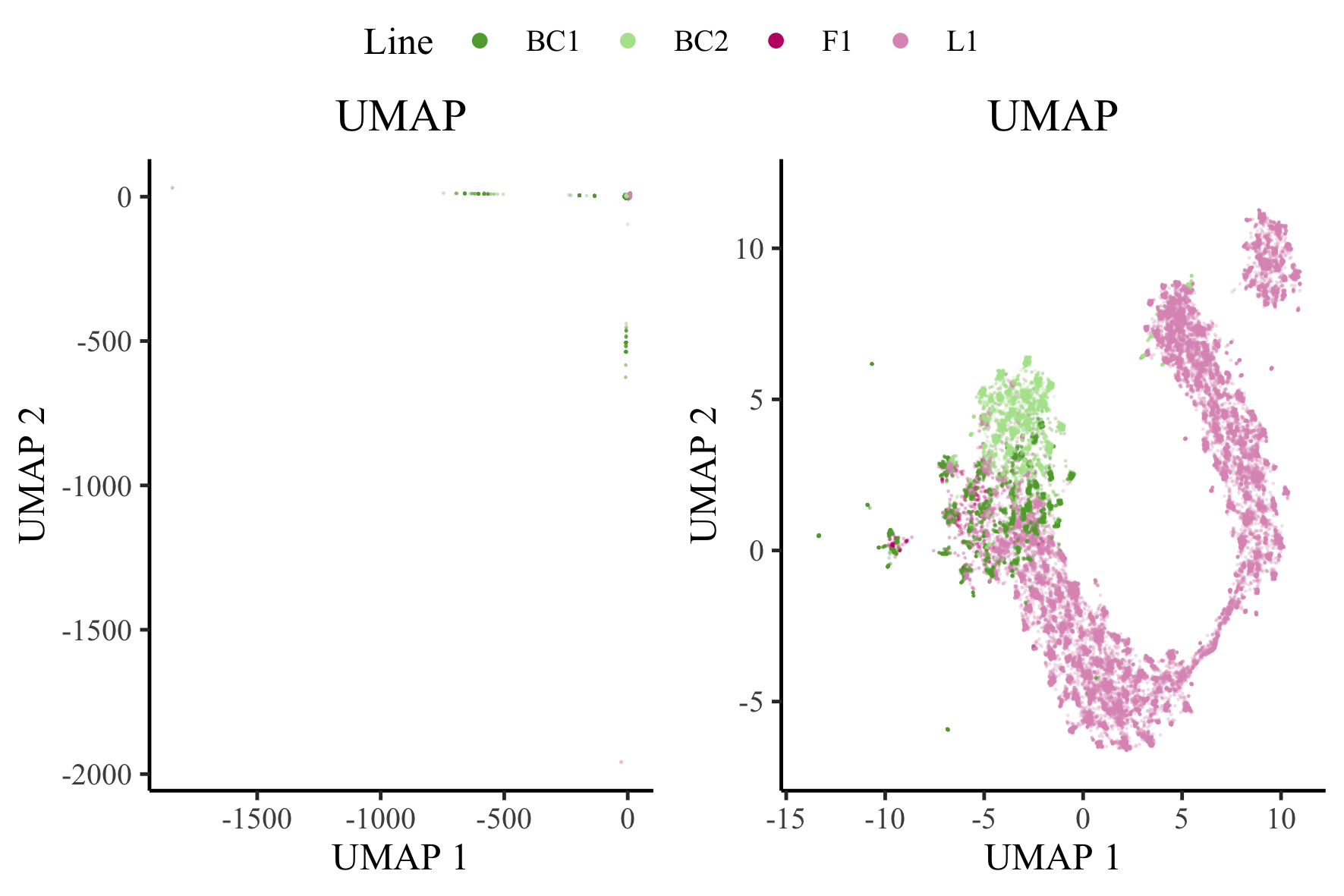

Supplement: Supplementary file 8 — Additional file 8. Visualisation of UMAP before and after cleaning the pig data. Here we visualise Uniform Manifold Approximation and Projection (UMAP) for genotyped purebred (L1), crossbred (F1), and backcross (BC1, BC2) pigs, before (a) and after (b) removing 378 (< 1%) pigs from the final UMAP plot for the sake of clarity. [file 12711_2022_767_MOESM8_ESM.png]
