# Supplementary material for: Optimisation of the core subset for the APY approximation of genomic relationships
Source: Genet Sel Evol. 2022 Nov 22;54:76. doi: 10.1186/s12711-022-00767-x (PMC9682752; doi:10.1186/s12711-022-00767-x)
Supplement: Supplementary file 3 — Additional file 3. Percentage of realised variation explained in G by each core subset in pigs. [file 12711_2022_767_MOESM3_ESM.docx]

**Additional File 3 (Table) - Percentage of realised variation explained in G by each core subset.**

| **Approach** | **Percentage of variation explained in G**^2^ | | | | | | | |
| --- | --- | --- | --- | --- | --- | --- | --- | --- |
|  | **10** | **30** | **50** | **70** | **90** | **95** | **98** | **99** |
| **Core animals** | **8** | **60** | **184** | **485** | **1658** | **2926** | **5546** | **8348** |
| **Random**^1^ | 0.02 | 0.12 | 0.37 | 0.97 | 3.33 | 5.88 | 11.14 | 16.76 |
| **Diagonal** | 0.02 | 0.17 | 0.50 | 1.28 | 4.18 | 7.18 | 13.17 | 19.39 |
| **Weighted**^1^ | 0.02 | 0.12 | 0.37 | 0.98 | 3.36 | 5.94 | 11.23 | 16.89 |
| **Conditional** | 0.02 | 0.16 | 0.47 | 1.18 | 3.79 | 6.48 | 11.96 | 17.75 |

^1^ For Random and Weighted core selection approaches mean over five replicates is shown; SD was always < 0.1 and is not shown.

^2^**G** is the genomic relationship matrix
