# Supplementary material for: Optimisation of the core subset for the APY approximation of genomic relationships
Source: Genet Sel Evol. 2022 Nov 22;54:76. doi: 10.1186/s12711-022-00767-x (PMC9682752; doi:10.1186/s12711-022-00767-x)
Supplement: Supplementary file 5 — Additional file 5. Bias for validation pigs as the regression coefficient from regression of their phenotypes (adjusted for the fixed effects) on their GEBV. [file 12711_2022_767_MOESM5_ESM.docx]

**Additional File 5 (Table) – Bias for validation pigs as the regression coefficient from regression of their phenotypes adjusted for the fixed effects on their GEBVs.**

| **Approach** | **Percentage of variation explained in G**^2^ | | | | | | | |
| --- | --- | --- | --- | --- | --- | --- | --- | --- |
|  | **10** | **30** | **50** | **70** | **90** | **95** | **98** | **99** |
| **Full** | 0.80 | 0.80 | 0.80 | 0.80 | 0.80 | 0.80 | 0.80 | 0.80 |
| **Random**^1^ | 1.08 (0.97) | 0.86 (0.13) | 0.70 (0.08) | 0.64 (0.10) | 0.76 (0.04) | 0.80 (0.02) | 0.80 (0.00) | 0.80 (0.00) |
| **Diagonal** | 0.67 | 0.62 | 0.60 | 0.62 | 0.75 | 0.79 | 0.80 | 0.81 |
| **Weighted**^1^ | 1.89 (1.23) | 0.97 (0.20) | 0.60 (0.14) | 0.65 (0.05) | 0.73 (0.03) | 0.80 (0.02) | 0.80 (0.00) | 0.80 (0.00) |
| **Conditional** | 0.72 | 0.67 | 0.73 | 0.69 | 0.80 | 0.79 | 0.79 | 0.80 |

^1^For Random and Weighted core selection approaches mean and SD (in parentheses) over five replicates is shown

^2^**G** is the genomic relationship matrix
