# Supplementary material for: Optimisation of the core subset for the APY approximation of genomic relationships
Source: Genet Sel Evol. 2022 Nov 22;54:76. doi: 10.1186/s12711-022-00767-x (PMC9682752; doi:10.1186/s12711-022-00767-x)
Supplement: Supplementary file 6 — Additional file 6. Number of preconditioned conjugate gradient rounds (PCG) to convergence in pigs. PCG convergence criterion was set to 10−12 and was run using the BLUP90IOD2 software executed in The University of Edinburgh High-Performance Computing environment. [file 12711_2022_767_MOESM6_ESM.docx]

**Additional File 6 (Table) - Number of preconditioned conjugate gradient rounds (PCG) to convergence in pigs.**

| **Approach** | **Percentage of variation explained in G**^2^ | | | | | | | |
| --- | --- | --- | --- | --- | --- | --- | --- | --- |
|  | **10** | **30** | **50** | **70** | **90** | **95** | **98** | **99** |
| **Random**^1^ | 38 (2) | 60 (1) | 85 (2) | 118 (3) | 216 (7) | 297 (1) | 415 (8) | 485 (4) |
| **Diagonal** | 42 | 60 | 107 | 169 | 349 | 505 | 726 | 834 |
| **Weighted**^1^ | 37 (1) | 60 (2) | 85 (2) | 115 (3) | 215 (4) | 303 (3) | 424 (9) | 496 (6) |
| **Conditional** | 45 | 63 | 87 | 127 | 242 | 326 | 441 | 578 |

^1^For Random and Weighted core selection approaches mean and SD (in parentheses) over five replicates is shown and rounded to the nearest integer

^2^**G** is the genomic relationship matrix
